# Aqueous Leaf Extract of *Jatropha gossypiifolia* L. (Euphorbiaceae) Inhibits Enzymatic and Biological Actions of *Bothrops jararaca* Snake Venom

**DOI:** 10.1371/journal.pone.0104952

**Published:** 2014-08-15

**Authors:** Juliana Félix-Silva, Thiago Souza, Yamara A. S. Menezes, Bárbara Cabral, Rafael B. G. Câmara, Arnóbio A. Silva-Junior, Hugo A. O. Rocha, Ivanise M. M. Rebecchi, Silvana M. Zucolotto, Matheus F. Fernandes-Pedrosa

**Affiliations:** 1 Laboratório de Tecnologia & Biotecnologia Farmacêutica (TecBioFar), Programa de Pós-graduação em Ciências Farmacêuticas (PPgCF), Universidade Federal do Rio Grande do Norte (UFRN), Natal, Rio Grande do Norte, Brazil; 2 Laboratório de Farmacognosia, Departamento de Farmácia, Universidade Federal do Rio Grande do Norte (UFRN), Natal, Rio Grande do Norte, Brazil; 3 Laboratório de Biotecnologia de Polímeros Naturais (BIOPOL), Programa de Pós-graduação em Bioquímica, Universidade Federal do Rio Grande do Norte (UFRN), Natal, Rio Grande do Norte, Brazil; 4 Laboratório de Hematologia Clínica, Departamento de Análises Clínicas e Toxicológicas, Universidade Federal do Rio Grande do Norte (UFRN), Natal, Rio Grande do Norte, Brazil; Universidade Federal do Rio de Janeiro, Brazil

## Abstract

Snakebites are a serious public health problem due their high morbi-mortality. The main available specific treatment is the antivenom serum therapy, which has some disadvantages, such as poor neutralization of local effects, risk of immunological reactions, high cost and difficult access in some regions. In this context, the search for alternative therapies is relevant. Therefore, the aim of this study was to evaluate the antiophidic properties of *Jatropha gossypiifolia*, a medicinal plant used in folk medicine to treat snakebites. The aqueous leaf extract of the plant was prepared by decoction and phytochemical analysis revealed the presence of sugars, alkaloids, flavonoids, tannins, terpenes and/or steroids and proteins. The extract was able to inhibit enzymatic and biologic activities induced by *Bothrops jararaca* snake venom *in vitro* and *in vivo*. The blood incoagulability was efficiently inhibited by the extract by oral route. The hemorrhagic and edematogenic local effects were also inhibited, the former by up to 56% and the latter by 100%, in animals treated with extract by oral and intraperitoneal routes, respectively. The inhibition of myotoxic action of *B. jararaca* reached almost 100%. According to enzymatic tests performed, it is possible to suggest that the antiophidic activity may be due an inhibitory action upon snake venom metalloproteinases (SVMPs) and/or serine proteinases (SVSPs), including fibrinogenolytic enzymes, clotting factors activators and thrombin like enzymes (SVTLEs), as well upon catalytically inactive phospholipases A_2_ (Lys49 PLA_2_). Anti-inflammatory activity, at least partially, could also be related to the inhibition of local effects. Additionally, protein precipitating and antioxidant activities may also be important features contributing to the activity presented. In conclusion, the results demonstrate the potential antiophidic activity of *J. gossypiifolia* extract, including its significant action upon local effects, suggesting that it may be used as a new source of bioactive molecules against bothropic venom.

## Introduction

Snakebites are a serious public health problem in many regions around the world, particularly in tropical and subtropical countries [1], [2]. The high morbi-mortality rate still has a great impact on the population and on health-care systems, especially in Africa, Asia, Oceania and Latin America and, unfortunately, public health authorities have given little attention to this problem [1]. Thus, snake envenomation is included in the 2009 World Health Organization (WHO) list of Neglected Tropical Diseases (NTDs) [3]. Conservative estimates indicate that, worldwide, there are more than 5 million snakebites, leading to 25,000–125,000 deaths [2], [3]. In Brazil, data from Ministry of Health shows that there are more than 25,000 snakebites per year [4].

More than 90% of the snakebites reported every year in Latin America are caused by *Bothrops* species [5]. In Brazil, the major representatives of the genus are *Bothrops jararaca*, *Bothrops alternatus*, *Bothrops atrox*, *Bothrops erythromelas*, *Bothrops jararacussu* and *Bothrops moojeni* [4]. Despite the existence of evident intraspecific and interspecific variations in the composition and biological activities of their venoms, bothropic venom can induce a qualitatively similar pathophysiological picture, characterized by immediate and prominent local tissue damage (including myonecrosis, hemorrhage and edema), cardiovascular alterations (especially hemorrhage and hypovolemic shock), coagulation disorders (most frequently blood incoagulability) and renal alterations (which could evolve into acute kidney injury) [5]. The snake envenoming is a complex pathophysiological process involving the simultaneous action of different types of toxins, such as snake venom serine proteinases (SVSPs), snake venom metalloproteinases (SVMPs), hyaluronidases and phospholipases A_2_ (PLA_2_) [5], [6].

Currently, the only available specific treatment is the antivenom serum therapy, which consists of a pool of neutralizing antibodies taken from serum of animals hyperimmunized against toxins of snake venoms. Its effectiveness consists of its ability to provide to the patient antibodies with a high affinity to snake venom, aiming to eliminate the toxins responsible for toxicity of the envenoming [7]. However, the antivenom has some disadvantages, such as limited effectiveness against local effects, risk of immunological reactions (including “serum sickness”), high cost and difficult access in some regions [5], [7]. If antivenom administration is initiated rapidly after envenomation, neutralization of systemic effects is usually achieved successfully. However, neutralization of local tissue damage is more difficult. This poor effectiveness against local effects, as well the increased time between accident and treatment are related to the temporary or permanent disability observed in many victims. It is estimated that 400,000 people are left with permanent disabilities after snakebites [3], [5].

In this context, the search for new complementary therapies to treat snakebites is relevant and medicinal plants could be highlighted as a rich source of natural inhibitors and pharmacologically active compounds [8], [9]. There are several reports of the popular use of medicinal plants against snake bites around the world, especially in tropical and subtropical regions such as Asia, Africa and South America [10], [11]. The main advantages of antiophidic plants are their low cost, easy access, stability at room temperature and ability to neutralize a broad spectrum of toxins, including the local tissue damage [9], [11].

*Jatropha gossypiifolia* L. (Euphorbiaceae) (**Figure S1**) is a medicinal plant popularly known in Brazil as “pinhão-roxo” or worldwide as “bellyache-bush”. It is largely used in folk medicine for various purposes, namely its uses as antiophidic, anti-inflammatory, anti-hemorrhagic, hemostatic and healing, among others [12]–[14]. Additionally, this species is included in the National List of Medicinal Plants of Interest to Brazilian Public Health System (*RENISUS*), which is a report published by the Brazilian Health Ministry that includes 71 species of medicinal plants that have the potential to generate pharmaceutical products of interest in the Brazilian public health system [15].

However, despite the widespread popular use of *J. gossypiifolia* as an antidote for snakebites, to best of our knowledge, no study has been found in the literature evaluating its antiophidic properties. Therefore, this study was carried out aiming to evaluate the antiophidic properties of the aqueous leaf extract of *J. gossypiifolia* against the enzymatic and biological activities induced by *B. jararaca* snake venom, and thus to evaluate the potentiality of the plant to obtain new natural alternatives for snakebite treatment. An aqueous extract was selected for this study and oral route *in vivo* was tested with the intention to simulate the most popular use of the plant, which is as a tea. Additionally, particular emphasis is given to inhibition of local effects of the venom.

## Materials and Methods

### Chemicals and reagents

Luteolin, orientin, isoorientin, vitexin, isovitexin, **D**-glicose, gallic acid, bovine serum albumin, 3-(4,5-dimethylthiazol-2-yl)-2,5-diphenyltetrazoliumbromide (MTT), azocasein, bovine fibrinogen, hexadecyltrimethylammonium bromide and *o*-dianisidine were purchased from Sigma-Aldrich (St. Louis, MO, USA). All reagents of sodium dodecilsuphate polyacrylamide gel electrophoresis (SDS-PAGE) were purchased from GE Healthcare (Piscataway, NJ, USA). All reagents used for cell culture procedures were purchased from Cultilab (Campinas, SP, Brazil). All other reagents and solvents used were of analytical grade. The water used was purified by reverse osmosis.

### Plant material

Leaves of *Jatropha gossypiifolia* L. (Euphorbiaceae) were collected in Rio Grande do Norte State, Brazil, at coordinates 36.80°W 5.27°S, in April 2012. The botanical identification of the material was performed by Msc. Alan de Araújo Roque and a voucher specimen was deposited at the Herbarium from the Centro de Biociências da Universidade Federal do Rio Grande do Norte, Brazil (UFRN 12561). The leaves were dried at room temperature, triturated and stored in hermetically sealed bottles away from light and humidity until use for extract preparation. The collection of the plant material was conducted under authorization of Brazilian Authorization and Biodiversity Information System (SISBIO) (process number 35017) and Brazilian Access Authorization and Dispatch Component of Genetic Patrimony (CGEN) (Process 010844/2013-9).

### Preparation of pool of plasma and red blood cell (RBC) suspension

After written informed consent had been obtained, blood from healthy adult volunteers who were free from medication for at least two weeks and fasted for 8 h was taken by venipuncture and collected into 0.105 M sodium citrate (9∶1 v/v, blood: citrate) or K_3_EDTA (1,5 mg EDTA: 1 mL blood) tubes (BD Vacutainer, Franklin Lakes, NJ, USA). A pool of plasma was prepared from the supernatants obtained after centrifugation at 800 g for 10 min at room temperature of the citrated blood and stored at −20°C until use. The plasma was used up to two weeks after being obtained.

For red blood cell (RBC) suspension preparation, blood collected with EDTA was centrifuged at 560 g for 10 min at room temperature and the red blood cell pellet was subsequently rinsed three times with phosphate buffer saline (PBS). A 20% (v/v) RBC suspension was obtained by dilution with PBS. The RBC was used immediately after preparation.

The procedures for human blood collection were approved by the Ethics Committee in Human Research from Universidade Federal do Rio Grande do Norte (protocol no. 092/09).

### Snake venom

The lyophilized *B. jararaca* snake venom was commercially purchased from Sigma-Aldrich (St. Louis, MO, USA) (product number V5625). The scientific use of the material was approved by the Brazilian Access Authorization and Dispatch Component of Genetic Patrimony (CGEN) (Process 010844/2013-9). The venom was weighed and dissolved in PBS.

### Animals

*Swiss* albino mice (30–35 g, 6–8 weeks-old) maintained under standard environmental conditions and fed with food and water *ad libitum* were used. All the procedures requiring animals were performed in agreement with the recommendations of the National Council for the Control of Animal Experimentation of Brazil (CONCEA) and the International Guiding Principles for Biomedical Research Involving Animals of the Council of International Organizations of Medical Sciences (CIOMS). The experimental protocols were approved by the Ethics Committee on Animal Use from Universidade Federal do Rio Grande do Norte (protocol no. 004/2013). On the day of the experiment, the animals were placed in the experimental room for at least one hour prior to tests for acclimation. The animals who received oral (p.o.) administration of extract were food fasted (only water *ad libitum*) 18 h prior to experiment. At the end of the experiments, the animals were euthanized by sodium thiopental overdose by intraperitoneal (i.p.) route.

### Aqueous leaf extract of *J. gossypiifolia* preparation

Dried leaves were submitted to decoction (10% w/v, plant: water) for 15 min at a temperature of around 100°C to obtain the aqueous leaf extract of *J. gossypiifolia* (yield: 13.57% relative to dry plant). The aqueous extract obtained after vacuum filtration was freeze-dried and dissolved in PBS at adequate concentrations for the biological assays.

### Phytochemical analysis of *J. gossypiifolia* aqueous leaf extract

For phytochemical analysis, the extract was fractionated by liquid-liquid partition with solvents of increasing polarity in order to obtain the dichloromethane (CH_2_Cl_2_), ethyl acetate (AcOEt), *n*-butanol (BuOH) and residual aqueous (RA) fractions. Then, the crude extract and fractions were analyzed by thin layer chromatography (TLC) using aluminum pre-coated sheets with silica gel F_254_ (Merck, Darmstadt, Germany) as adsorbent. Three different mobile phases were employed: (1) ethyl acetate: formic acid: water (8∶1∶1 v/v/v), (2) toluene: ethyl acetate: formic acid (5∶5∶0.5 v/v/v) and (3) *n*-butanol: acetic acid: water (3∶1∶1 v/v/v). The chromatograms were analyzed under 254 and 365 nm UV light and then sprayed with specific chromogenic agents according to the class of compounds investigated (sulfuric vanillin + heating, natural reagent A, ferric chloride, Dragendorff reagent and ninhindrin + heating). The retention factors (*Rf*), color and behavior of the spots were compared with chromatographic profiles of reference substances in the literature [16]. Standard samples of flavonoids were employed for co-TLC analysis.

Additionally, the total content of sugar, phenolic compounds and protein in the crude extract were quantified. Total sugar was estimated by Dubois method using **D**-glicose as standard [17]. Phenolic compounds were determined by Folin-Ciocalteu's colorimetric method using gallic acid as standard [18]. The Bradford method was used for protein quantification using albumin as standard [19].

### Hemolytic assay

The hemolytic assay of aqueous leaf extract from *J. gossypiifolia* was performed as previously described in literature, with a few modifications [20]. Briefly, 5 µL of 20% (v/v) red blood cell (RBC) suspension were incubated at 37°C for 60 min with 500 µL of extract at different concentrations (0.1–2 µg/µL). The mixtures were then centrifuged at room temperature for 2 min at 8,600 g and the absorbance of the supernatant was measured at 540 nm in a microplate reader (Epoch-Biotek, Winooski, VT, USA). Water was used as positive control (100% RBC lysis) and PBS as negative control (absence of RBC lysis). The values of treated cells were calculated as a percentage of the positive control.

### *In vitro* cytotoxicity assay

Human embryonic kidney 293 cells (HEK-293) (ATCC CRL-1573, Manassas, VA, USA) were cultured under standard conditions in Dulbecco's modified Eagle's medium (DMEM) supplemented with fetal bovine serum (FBS). Cells were maintained in cell culture flasks at 37°C in a humidified atmosphere containing 5% CO_2_ and were collected by treatment with trypsin. Cells (1×10^4^ cells per well) were seeded in complete medium and cultured for 24 h in 96 well microplates to promote adhesion. The following day, the medium was removed and replaced with fresh medium free of FBS. Then the next day, the medium was replaced with fresh medium with FBS containing serial dilutions of the extract (3.9–500 µg/mL) previously sterilized in a 0.45 µm membrane. The negative control was exposed to the standard medium without sample. After 24 h, the 3-(4,5-dimethylthiazol-2yl)-2,5-diphenyltetrazolium bromide (MTT) assay was performed as a marker of cell viability, as previously described in literature [21]. Briefly, medium containing extract was replaced with medium containing 1 mg/mL of MTT and incubated for 4 h at 37°C. After incubation, the supernatant was removed and the purple formazan crystal formed was solubilized in ethanol, stirred for 15 min at room temperature and the absorbance was measured at 570 nm in a microplate reader (Epoch-Biotek, Winooski, VT, USA). The absorbance of the negative control (no extract) was considered as 100% cell viability and the values of treated cells were calculated as a percentage of the negative control.

### Inhibition of proteolytic activity upon azocasein

The proteolytic activity of *B. jararaca* venom was determined using azocasein (1% w/v, in 50 mM Tris-HCl pH 7.5) as substrate, as previously described in literature, with a few modifications [22]. Venom aliquots pre-incubated for 60 min at 37°C were mixed with 2.5 mM HCl and azocasein and incubated for 120 min at 37°C. The enzymatic reaction was stopped by adding trichloroacetic acid (20% w/v). After 30 min standing at room temperature, the tubes were centrifuged at 8,600 g for 10 min. The supernatant was removed and mixed with 2 M NaOH. 10 min later, tubes were read at 440 nm in an UV-VIS spectrophotometer (Evolution 60S, Waltham, MA, USA). Blanks for each concentration were prepared the same way, except by adding the substrate only after trichloroacetic acid addition. One Minimum Azocaseinolytic Concentration (MAC) was defined as the amount of venom able to produce a variation in absorbance of 0.200 at 440 nm. For inhibition assay, one MAC of venom was pre-incubated for 60 min at 37°C with different concentrations of *J. gossypiifolia* aqueous leaf extract (1∶1–1∶100 venom: extract, w/w), and then the proteolytic activity was measured, as described above. Venom alone was used as control (100% of proteolytic activity), as well as extract alone.

### Inhibition of fibrinogenolytic activity

The fibrinogenolytic activity of *B. jararaca* venom was determined as previously described in literature, with a few modifications [23]. 10 µg of venom was pre-incubated for 60 min at 37°C with different concentrations of *J. gossypiifolia* aqueous leaf extract (1∶1–1∶100 venom: extract, w/w). Then, 50 µg of fibrinogen was added and incubated for 180 min at 37°C. The enzymatic reaction was stopped by adding 1.5 M Tris-HCl pH 8.8 containing 10% v/v glycerol, 10% v/v β-mercaptoetanol, 2% w/v SDS and 0.05% w/v bromophenol blue, followed by boiling for 5 min. The samples were then analyzed by 12% SDS-PAGE [24]. Venom alone was used as control (100% of proteolytic activity). Fibrinogen alone was also used as control, for visualization of the intact fibrinogen profile.

Additionally, the fibrinogenolytic activity of *B. jararaca* was tested by zymography, as previously described in literature, with a few modifications [25]. 12% SDS-PAGE were prepared and polymerized at final concentration of 1 mg/mL of fibrinogen. 5 µg of venom was pre-incubated for 60 min at 37°C with different concentrations of *J. gossypiifolia* aqueous leaf extract (1∶1–1∶25 venom: extract, w/w). Then, the samples were treated with Laemmli buffer in the absence of reducing agents, followed by room temperature incubation for 5 min. After electrophoresis, gels were washed 3 times for 30 min with Triton X-100 to remove SDS and then were washed one time, for 30 min, in 50 mM Tris-HCl pH 7.4 containing 200 mM NaCl and 5 mM CaCl_2_. After washing, the buffer was replaced with a fresh one and the gels were incubated for 24 h at 37°C in this buffer. After 3 washes with water for 5 min, the gels were stained with 0.5% Coomassie Brilliant Blue. Clear zones of substrate lysis against a blue background stain indicated presence of fibrinogenolytic enzymes. Venom alone was used as control (100% of proteolytic activity), as well as extract alone. Molecular mass marker was used to estimate the approximate molecular mass of the fibrinogenolytic enzymes.

### Inhibition of procoagulant activity

The procoagulant activity of *B. jararaca* venom was determined on fibrinogen solution (6 mg/mL) and plasma, as previously described in literature, with a few modifications [26]. 100 µL of fibrinogen or plasma were incubated at 37°C for 2 min. Then, venom aliquots pre-incubated for 60 min at 37°C were added to fibrinogen or plasma and the clotting time recorded by a digital coagulometer (“Laser Sensor” Clotimer, CLOT, São Paulo, SP, Brazil). One Minimum Coagulant Concentration was defined as the amount of venom able to clot fibrinogen (MCC-F) or plasma (MCC-P) in 120 s. For inhibition assay, one MCC-F or MCC-P of venom was pre-incubated for 60 min at 37°C with different concentrations of *J. gossypiifolia* aqueous leaf extract (1∶0.5–1∶10 venom: extract, w/w), and then the clotting time was measured as described above. Venom alone was used as control (100% of coagulant activity), as well as extract alone.

Additionally, the anticoagulant activity of the extract, in absence of venom, was determined using the activated partial thromboplastin time (aPTT) and prothrombin time (PT) tests, as previously described in literature, with a few modifications [27]. The test was carried out using commercial reagent kits (CLOT Bios Diagnostica, São Paulo, SP, Brazil). For aPTT test, plasma (90 µL) was mixed with 10 µL of extract at different concentrations in PBS (0.1–2 µg/µL) and incubated at 37°C for 5 min at 37°C, before the addition of pre-warmed (37°C) aPTT reagent (rabbit brain extract and ellagic acid) and incubation at 37°C for 2 min. Pre-warmed (37°C) 25 mM calcium chloride was then added and the clotting time recorded by a digital coagulometer. For PT test, plasma (90 µL) was mixed with 10 µL of extract at different concentrations (0.1–2 µg/µL) and incubated at 37°C for 5 min. Then, 200 µL of PT assay reagent (rabbit brain extract and calcium chloride) pre-warmed at 37°C for 10 min was added and the clotting time was recorded. In both tests, plasma alone was used as control (absence of anticoagulant activity).

### Antioxidant activity evaluation

The *in vitro* antioxidant activity of the aqueous leaf extract of *J. gossypiifolia* was performed using different models (total antioxidant capacity, copper and iron chelation, hydroxyl and superoxide radicals scavenging, and reducing power), as previously described in literature [28]. The IC_50_ (concentration that presented 50% of the referred activity) was calculated.

### Inhibition of phospholipase activity

The phospholipase activity of *B. jararaca* venom was determined using an egg yolk suspension (1∶3 v/v, egg yolk: PBS) as substrate, as previously described in literature, with a few modifications [29]. Medium containing 1% (w/v) agarose, 10% (v/v) egg yolk suspension and 0.1 mM calcium chloride was prepared in PBS and placed in Petri dishes until solidification. After becoming solidified, holes of 0.5 mm were produced in the medium for application of samples. Venom aliquots pre-incubated for 60 min at 37°C were applied in the medium and incubated for 24 h at 50°C. Clear zones of substrate lysis (halos) against a yellow background indicated presence of phospholipase activity. The halo diameter was measured with a digital caliper (Digimess, São Paulo, SP, Brazil). One Minimum Phospholipase Concentration (MPC) was defined as the amount of venom able to produce a phospholipase halo of 25 mm. For inhibition assay, one MPC of venom was pre-incubated for 60 min at 37°C with different concentrations of *J. gossypiifolia* aqueous leaf extract (1∶1–1∶100 venom: extract, w/w), and then the phospholipase activity was measured as described above. Venom alone was used as control (100% of phospholipase activity), as well as extract alone.

### Inhibition of defibrinogenating activity

The defibrinogenating activity of *B. jararaca* venom was performed using the *in vivo* model of blood incoagulability, as previously described in literature, with a few modifications [26]. Groups of 4 animals were treated with different doses of *J. gossypiifolia* extract (50, 100 and 200 mg/kg, in a volume of 10 mL/kg of body weight) by gavage (p.o.). After 60 min, the animals received by intraperitoneal (i.p.) route 10 µg of venom (200 µL). 4 h later, the animals were anesthetized with sodium thiopental and the blood was collected. The blood was placed in microtubes and left standing at room temperature for 60 min. The presence or absence of clotting formation after this period was registered. A group where animals received i.p. injection of venom and p.o. treatment of PBS was used as control and considered as 100% of defibrinogenating activity (absence of clot formation). Another group that received i.p. injection and p.o. treatment of PBS was used as negative control (absence of defibrinogenating activity, positive clot formation).

### Inhibition of local hemorrhagic activity

The hemorrhagic activity of *B. jararaca* venom was performed using the *in vivo* model of local hemorrhage, as previously described in literature, with a few modifications [30]. Groups of 5 animals were treated with different doses of *J. gossypiifolia* extract (50, 100 and 200 mg/kg, in a volume of 10 mL/kg of body weight) p.o. After 60 min, the animals received a subcutaneous (s.c.) injection of 25 µg of venom (100 µL) in the dorsal region. 3 h later, the animals were sacrificed and had the inner surface of the skin exposed. After photo documentation of the hemorrhagic halos produced, the hemorrhagic skin was removed, weighted, fragmented and homogenized with water (1 mL of water for each 100 mg of tissue). The samples were sonicated for 30 min in ice bath and then incubated for 48 h at 4°C for hemoglobin extraction. The supernatant obtained after centrifugation at 800 g for 30 min was read at 540 nm in an UV-VIS spectrophotometer (Evolution 60S, Waltham, MA, USA) for hemoglobin content quantification, considered as a marker of hemorrhage. A group where animals received s.c. injection of venom and p.o. treatment of PBS was used as control and considered as 100% of hemorrhage activity. Another group that received s.c. injection and p.o. treatment of PBS was used as negative control (absence of hemorrhagic halo).

### Inhibition of edematogenic activity

The edematogenic activity of *B. jararaca* venom was evaluated using the *in vivo* model of paw edema as previously described in literature with a few modifications [31]. Groups of 5 animals were treated with different doses of *J. gossypiifolia* extract (50, 100 and 200 mg/kg, in a volume of 10 mL/kg of body weight) p.o. or i.p. route. After 60 min, the animals received an intraplantar (i.pl.) injection of 3 µg of venom (50 µL) in the right hind paw. The individual right hind paw thickness was further measured at 0, 30, 60, 90 and 120 min post injection with a digital caliper (Digimess, São Paulo, SP, Brazil). Edema was expressed as the percentage difference between the thickness of the paw after (at respective times) and before (basal values) venom injection. A group of animals that received i.pl. injection of venom and p.o. or i.p. treatment of PBS was used as control and considered as 100% of edematogenic activity. Another group that received i.pl. injection and p.o. or i.p. treatment of PBS was used as negative control (absence of edematogenic activity).

Moreover, at the end of the experiment, the animals were sacrificed and their right hind paws were removed and analyzed for myeloperoxidase (MPO) activity, as previously described in literature, with a few modifications [32]. Tissues were homogenated in 0.5% hexadecyltrimethylammonium bromide buffer (1 mL of buffer for each 50 mg of tissue), sonicated in ice bath for 3 min, submitted at 3 cycles of freeze-thaw and finally sonicated for 3 min once more, for MPO enzyme extraction. 10 µL of the supernatant obtained after centrifugation at 1,400 g for 15 min at 4°C was mixed with 50 mM potassium phosphate pH 6.0 containing 0.0005% hydrogen peroxide and 0.167 mg/mL *o*-dianisidine. The MPO activity was colorimetrically determined using a microplate reader (Epoch-Biotek, Winooski, VT, USA), analyzing changes in absorbance at 460 nm for 5 min. The left hind paws (which did not received any treatment) from venom control group were used as control (basal values). The right paws of groups that received i.pl. injection of venom and p.o. or i.p. treatment of PBS were used as control and considered as 100% of MPO activity.

### Inhibition of myotoxic activity

The myotoxic activity of *B. jararaca* venom was performed using the serum creatine kinase (CK) level as a quantitative marker of miotoxicity, as previously described in literature, with a few modifications [33]. Groups of 5 animals were treated with different doses of *J. gossypiifolia* extract (50, 100 and 200 mg/kg, in a volume of 10 mL/kg of body weight) p.o. or i.p. route. After 60 min, all the animals received an intramuscular (i.m.) injection of 50 µg of venom (50 µL) in the left thigh. 3 h later, the animals were anesthetized with sodium thiopental and the blood was collected. The blood samples were incubated for 10 min at 37°C and then centrifuged at 8,600 g for 10 min to obtain the serum. The serum CK activity was determined using a commercial kit (Bioclin, Belo Horizonte, MG, Brazil) according to manufacturer's protocol adapted for reading in microplate reader (Epoch-Biotek, Winooski, VT, USA). A group of animals received i.m. injection of venom and p.o. or i.p. treatment of PBS was used as control and considered as 100% of myotoxic activity. Another group that received i.m. injection and p.o. or i.p. treatment of PBS was used as negative control (absence of miotoxicity).

### Interaction between extract and proteins

The proteolytic action of the aqueous leaf extract from *J. gossypiifolia* was assessed on albumin and *B. jararaca* venom proteins, as previously described in literature, with a few modifications [34]. Fixed amount of albumin or venom, with different concentrations of extract (1∶1–1∶100 albumin or venom: extract, w/w), were incubated for 60 min at 37°C and analyzed by SDS-PAGE 12% under reducing and non-reducing conditions [24], for comparison with the proteins alone to analyze possible changes in electrophoretic pattern (e.g., vanishing of bands with appearance of degradation products).

Additionally, the protein precipitating action of the extract was evaluated using the Bradford dye-protein binding assay, as previously described in literature, with a few modifications [35]. Briefly, a fixed amount of albumin or venom was incubated with different concentrations of extract for 60 min at 37°C. The supernatant obtained after centrifugation at 5,000 g for 15 min was mixed with Bradford reagent (0.01% Coomassie Brilliant Blue, 4.7% ethanol and 8.5% phosphoric acid) and read at 595 nm after 5 min of standing (“ABS test”) in an UV-VIS spectrophotometer (Evolution 60S, Waltham, MA, USA). The absorbances obtained were compared with tubes containing only protein (“ABS control”). Tubes containing the same amount of extract, in absence of protein, were used as blanks (“ABS blank”). The percentage of protein precipitation (%PPT) was calculated as follows: 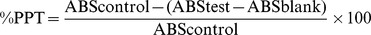


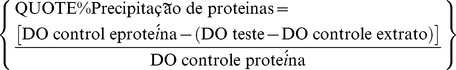



### Statistical analysis

All results are presented as mean ± standard error of mean (SEM). One-way ANOVA with Tukey's post test and regression analysis were performed using GraphPad Prism version 5.00 (San Diego, CA, USA). *p* values less than 0.05 were considered significant.

## Results and Discussion

### Phytochemical analysis of *J. gossypiifolia* aqueous leaf extract

The phytochemical analysis of *J. gossypiifolia* aqueous leaf extract was performed by TLC. But first, the crude extract was fractionated by liquid-liquid partition to obtain fractions with different polarities and thus facilitating the chromatographic analysis of the compounds.

To the best of our knowledge, there are no phytochemical studies regarding the use of water as solvent for the extraction of *J. gossypiifolia* constituents. This is important to be noted since popular use occurs more frequently with infusions or decoctions, and thus, little is known about the constitution of this kind of extract. Additionally, more commonly, the studies found in the literature use solvents or mixtures of solvents with non polar characteristic, which could contribute to further characterization of non-polar compounds, such as terpenoids and lignoids. Isolation of polar compounds such as flavonoids, tannins and sugars are poorly described regarding the species so far [14], [36].

By TLC analysis with specific spray reagents, spots suggestive of the presence of alkaloids, terpenes and/or steroids, phenolic compounds, flavonoids, tannins and amines were observed. By co-TLC analysis, when considering the color developed by natural reagent A (flavonoid specific reagent), *Rf* and spot overlap with standard, it was possible to suggest the presence of the flavonoids orientin, isoorientin, luteolin, vitexin and isovitexin in the extract. With the exception of luteolin, the other flavonoids have already been identified in the leaves of *J. gossypiifolia* [37], [38]. For the genus *Jatropha*, luteolin was described previously only for the species *Jatropha unicostata* [39].

Additionally, the content of sugar, phenolic compounds and proteins quantified were 20.0±0.3, 18.7±1.5 and 2.4±0.4%, respectively. So, the presence of phenolic compounds and sugars could be confirmed and it could be visualized that proteins represent only a small percentage of the crude extract composition.

The presence of flavonoids and tannins could be especially interesting in antiophidic plants since flavonoids are able to promote strong hydrogen bonds with amides of protein chains and exhibit metal chelating activity and tannins have the ability to precipitate proteins [8]. The flavonoid luteolin, for example, as previously described in literature, presents inhibitory action against hyaluronidases from *Crotalus adamenteus* snake venom [40].

### Cytotoxicity assays of *J. gossypiifolia* aqueous leaf extract

Keeping in mind that *Jatropha* species are known to be toxic [41] and considering previous studies that showed that ethanol extracts from *J. gossypiifolia* aerial parts exhibited noticeable toxicity, *in vitro* cytotoxicity studies were performed as a preliminary way to evaluate the potential toxicity of the aqueous leaf extract of *J. gossypiifolia* employed in the present work.

In both cytotoxicity models used (hemolytic activity and cytotoxicity against HEK-293 cells), the extract did not show cytotoxicity (results not shown), in any concentration tested (concentrations up to 2 µg/µL). It is important to note that both RBC and HEK-293 are human cells, which can reinforce the present observation. These results may suggest that the aqueous extract of the leaves, compared to the ethanol extract of the aerial parts tested by Mariz et al. [42], may be less toxic possibly due to an eventual difference in chemical composition that may have occurred, taking into account both the different plant parts and extractor solvent used for the different preparations of the extracts. In fact, a study investigating the acute oral toxicity of an aqueous leaf extract of *J. gossypiifolia* showed no sign of toxicity in rats in doses up to 2,000 mg/kg [43]. However, it is important to note that the toxicity of the aqueous leaf extract of *J. gossypiifolia* should not be entirely discarded and further studies are still needed to ensure its safety.

### Antiophidic activity evaluation of *J. gossypiifolia* aqueous leaf extract

Plants from *Jatropha* genus are frequently associated with antiophidic properties [44]. However, studies that evaluate the antiophidic activity of *Jatropha* species are very scarce in the literature. Only some studies have shown the antiophidic properties of *Jatropha elliptica* and *Jatropha mollissima,* with interesting results, according to the venom tested [45], [46]. However, studies showing the antiophidic activity of *J. gossypiifolia* were not found. In view of this and the popular use of the plant, this species was chosen as object of this study.

SVSPs and SVMPs are key pieces in *Bothrops* poisoning, especially regarding the hemostatic effects [5], [47]. Thus, inhibition of proteolytic enzymes is important when thinking about molecules with antiophidic activity. Snake venoms are rich in several proteolytic enzymes that degrade a wide variety of natural substrates, such as casein, fibrinogen and collagen, among others. It is known that several hemorrhagic and defibrinogenating toxins in snake venoms show significant activity against these substrates [48].

**Figure 1:**
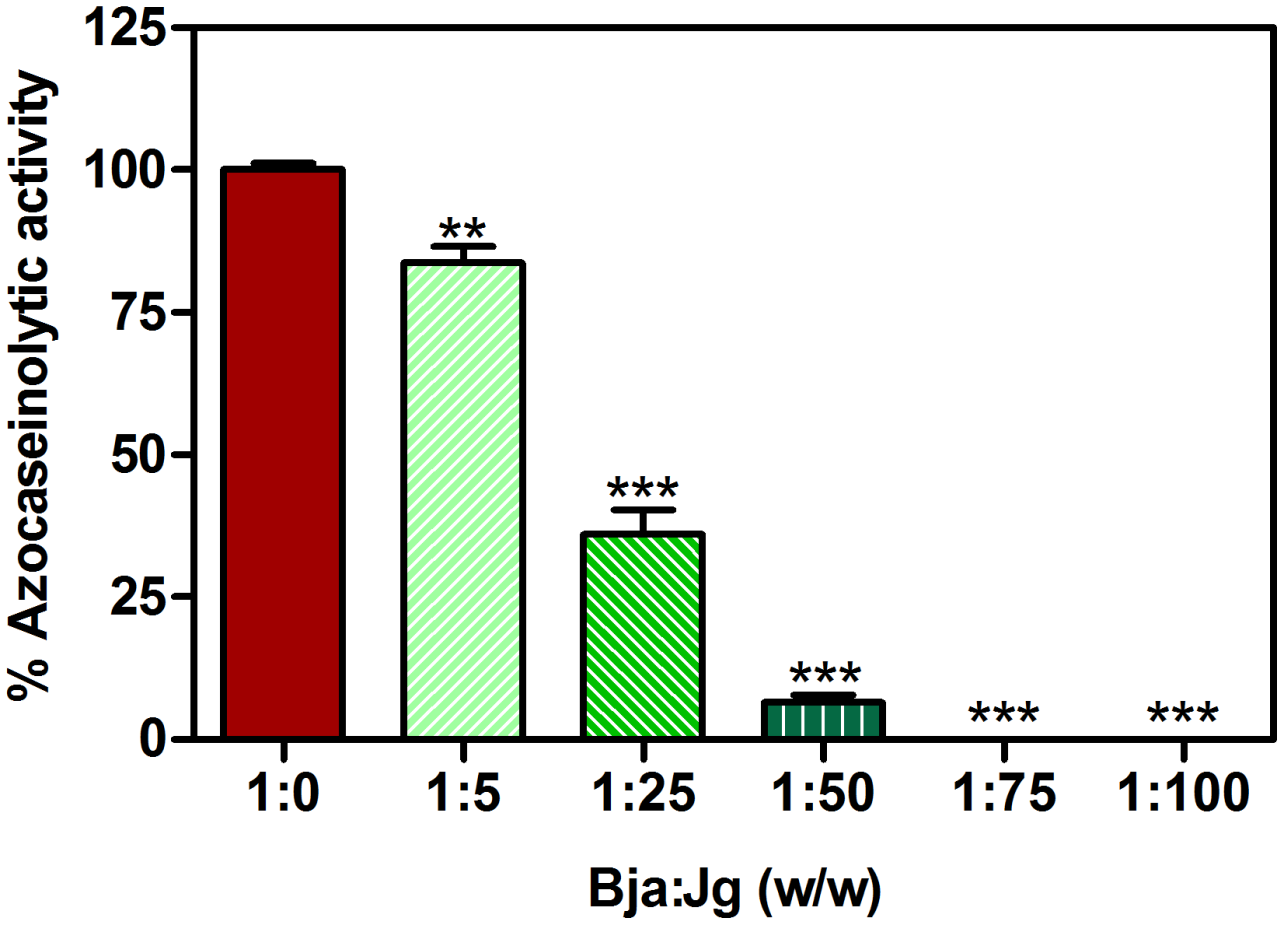
Inhibition of azocaseinolytic activity of *B. jararaca* by aqueous leaf extract of *J. gossypiifolia*. *B. jararaca* (Bja) venom, pre-incubated for 60 min at 37°C alone (control) or with different ratios of aqueous leaf extract of *J. gossypiifolia* (Jg) (w/w) (test), was incubated with azocasein for 2 h at 37°C. The reaction was stopped with tricloroacetic acid and the supernatant (product of reaction) obtained mixed with equal volume of NaOH was read at 440 nm. Blanks for each concentration were prepared the same way, except by adding azocasein only after trichloroacetic acid addition. The percentage of activity was calculated as: [(ABStest – ABSblank) ÷ ABScontrol]×100. Values expressed as mean±SEM with n = 3. **p<0.01 and ***p<0.001 when compared to control (1∶0 w/w, Bja:Jg) by Tukey's test (ANOVA).

Consequently, one of the first tests performed in this study was the inhibition of proteolytic activity of *B. jararaca* venom. The results obtained in this study revealed that the extract efficiently inhibited the venom proteolytic activity on azocasein, inhibiting completely the activity at higher concentrations (**Figure 1**). This result indicates a significant inhibitory action upon SVSPs and/or SVMPs.

The blood incoagulability produced by *Bothrops* envenomation is associated with the combined action of diverse toxins, such as fibrinogenolytic enzymes, snake venom thrombin like enzymes (SVTLEs) and clotting factors activators, which are all proteases [47], [49]. The inhibition of these toxins could contribute for the inhibition of the blood incoagulability scenario. So, in view of the anti-proteolytic activity presented by the extract, the possible inhibitory role in blood incoagulability was investigated.

**Figure 2:**
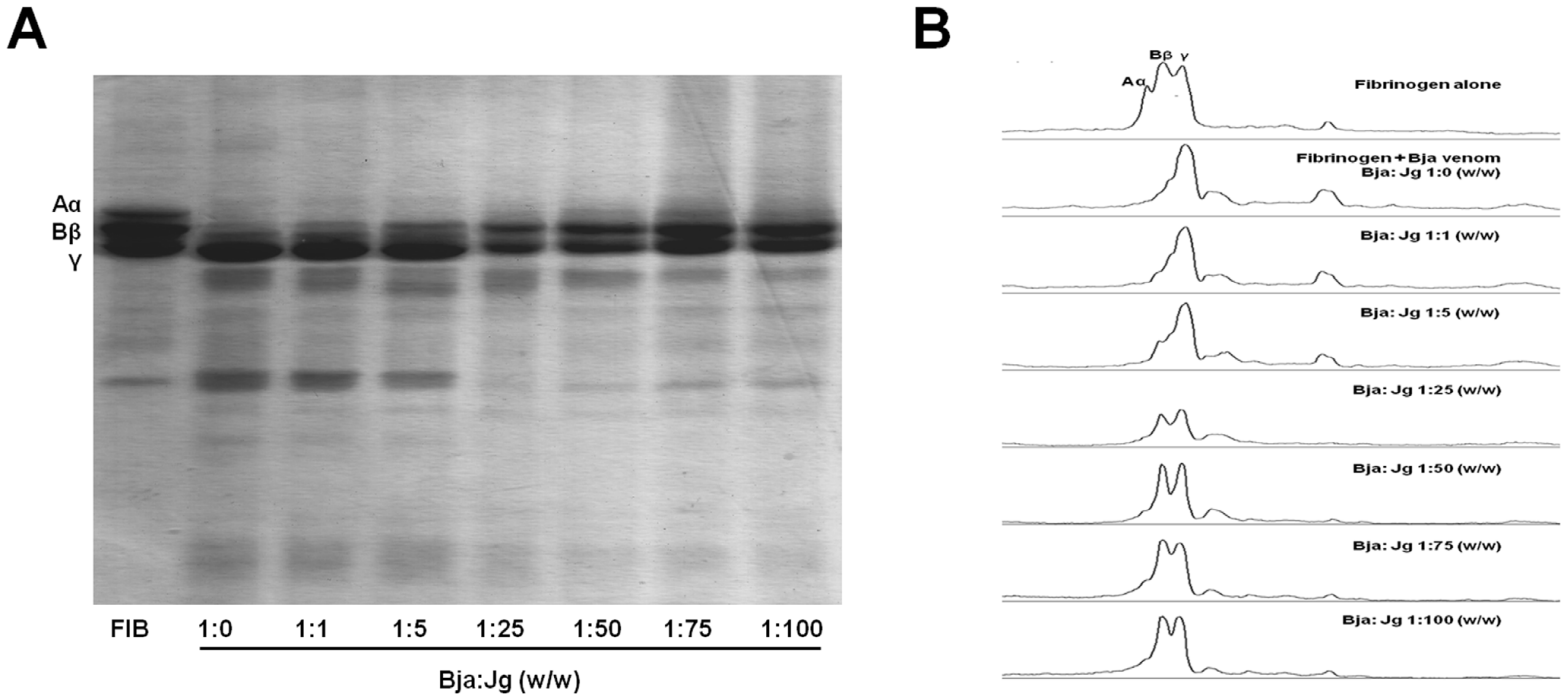
Inhibition of fibrinogenolytic activity of *B. jararaca* by aqueous leaf extract of *J. gossypiifolia*. *B. jararaca* (Bja) venom, pre-incubated for 60 min at 37°C alone (control) or with different ratios of aqueous leaf extract of *J. gossypiifolia* (Jg) (w/w), was incubated with fibrinogen for 3 h at 37°C. The reaction was stopped with appropriate buffer followed by boiling for 5 min. The samples were then analyzed by SDS-PAGE 12% and stained with Coomassie Brilliant Blue G-250. A: SDS-PAGE gel. FIB: fibrinogen alone (control). B: densitography analysis of the respective SDS-PAGE gel lines presented in A.

Fibrinogenolytic enzymes are toxins that directly split off fragments mostly from the C terminal regions of Aα, Bβ and γ chains of fibrinogen molecule, rendering it unclottable by thrombin. These enzymes do not convert fibrinogen to fibrin and the produced fibrinogen degradation products usually differ from those produced by plasmin. Fibrinogenolytic enzymes exert defibrinogenating action *in vivo* by consuming the circulant fibrinogen, contributing to the consumption coagulopathy. Since the fibrinogen levels are rapidly reduced, the patient tends to present blood incoagulability and prolonged clotting time [49], [50]. As could be observed in **Figure 2**, the aqueous leaf extract of *J. gossypiifolia* was able to inhibit fibrinogenolytic enzymes from *B. jararaca*. The extract, at higher concentrations, protected the fibrinogen from the proteolytic action from venom, protecting its Aα and Bβ chains. In presence of crescent amount of extract, can be observed a gradual reappearance of the fibrinogen chains (especially the Bβ chain), as well as a progressive disappearing of the fibrinogen degradation products produced by the venom proteolytic action. By zymography experiments using fibrinogen as substrate, the inhibition of fibrinogenolytic activity was confirmed and it could be observed that the extract inhibits, preferentially, different *B. jararaca* fibrinogenolytic enzymes of about 26 and 28 kDa (results not shown).

SVTLEs are SVSPs that clot fibrinogen *in vitro*, converting fibrinogen into fibrin. However, *in vivo*, these toxins cause a consumption coagulopathy similar to disseminated intravascular coagulation, leading to blood incoagulability. These toxins, unlike thrombin, did not activate FXIII (clotting factor fibrin stabilizer), which results in formation of abnormal fibrin clots composed of short polymers not stabilized by transglutaminase-catalyzed crosslinking. So, the fibrinolytic system quickly degrades such clots, contributing to the defibrinogenating action observed [49]-[51]. According to our results, based on the inhibition of procoagulant activity of *B. jararaca* venom upon fibrinogen by the aqueous leaf extract of *J. gossypiifolia* (**Table 1**), an inhibition of SVTLEs could be suggested.

**Table 1:** Inhibition of procoagulant activity of *B. jararaca* by aqueous leaf extract of *J. gossypiifolia*.

Bja:Jg (w/w)	Clotting time (s)	
	Upon fibrinogen	Upon plasma
**0∶0 (PBS)**	>240	>240
**1∶0**	123.8±1.1	121.4±1.7
**1∶0.5**	128.7±0.3	235.0±5.0***
**1∶1**	144.9±1.8**	227.3±12.7**
**1∶2**	150.2±5.5***	NT
**1∶10**	NT	>240***

Bja: *B. jararaca* venom. Jg: aqueous leaf extract of *J. gossypiifolia.* NT: not tested.

Values expressed as mean±SEM with n = 3. **p<0.01 and ***p<0.001 when compared to control (1∶0 w/w, Bja:Jg) by Tukey's test (ANOVA).

The inhibition of procoagulant activity of *B. jararaca* venom upon plasma was also evaluated, since this test, along with SVTLEs, also evaluates the action of clotting factor activators toxins. These toxins also present procoagulant activity *in vitro*, but acting on the activation of some clotting factors, such as FX and prothrombin [47]. As can be observed in **Table 1**, the extract efficiently inhibited the procoagulant action of the venom upon plasma too. Although inhibition of clotting activity on fibrinogen was not complete, it was observed that the anticoagulant activity of the plasma was maintained for up to 10 min after the addition of the venom. This could suggest that the extract could also inhibit the effect of other procoagulant toxins rather than only SVTLEs.

**Figure 3:**
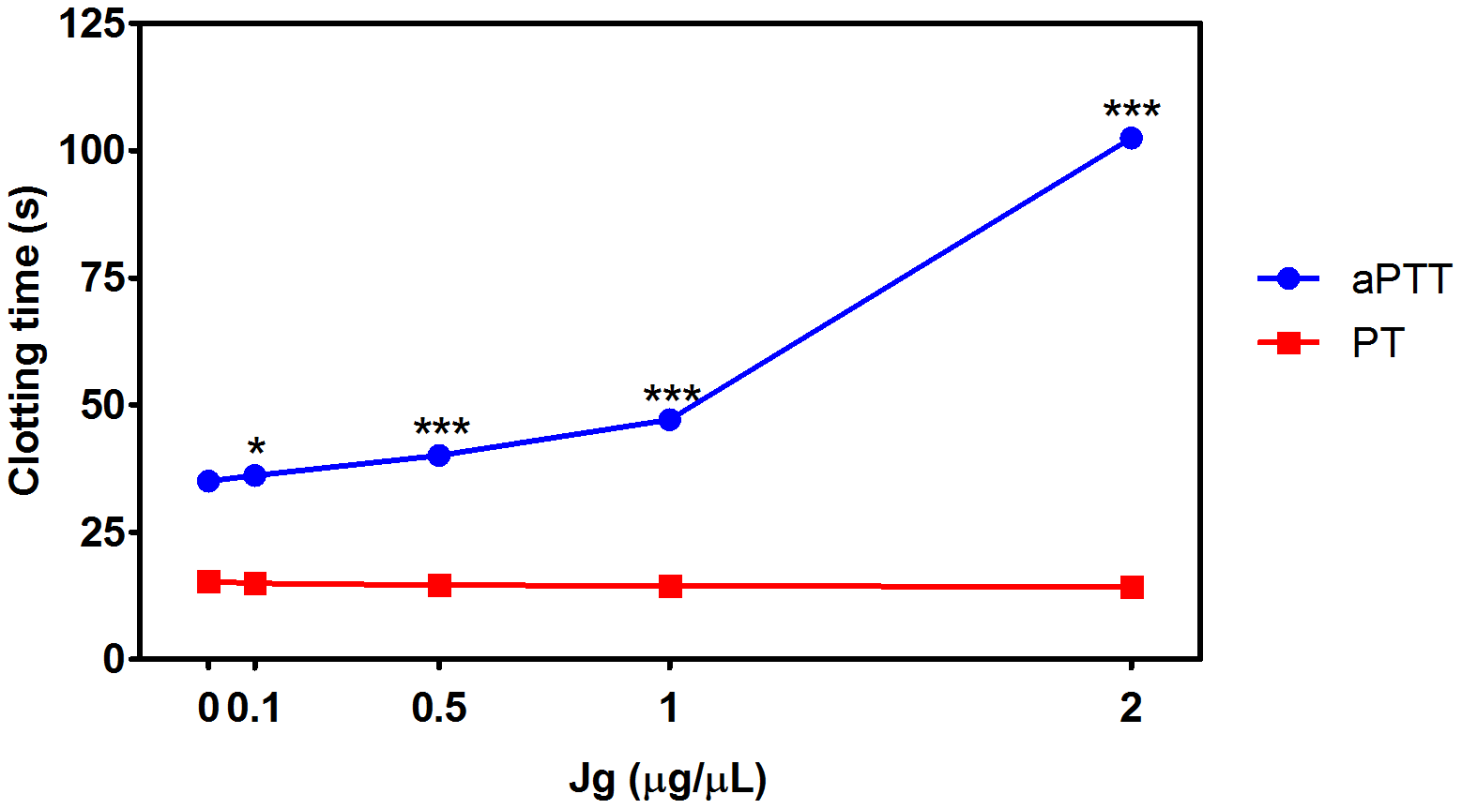
Anticoagulant activity of aqueous leaf extract of *J. gossypiifolia*. Different concentrations of aqueous leaf extract of *J. gossypiifolia* (Jg) were incubated with human citrated plasma for 5 min. After that, activated partial thromboplastin time (aPTT) and prothrombin time (PT) tests were performed using commercial kits according to manufacturer's protocol. Values expressed as mean±SEM with n = 3. *p<0.05 and ***p<0.001 when compared to control (absence of Jg) by Tukey's test (ANOVA).

In view to analyze if the anticoagulant activity observed in the extract was due to inhibition of toxins or if there was also an anticoagulant action upon endogenous factors, aPTT and PT tests with the extract were performed, in absence of venom. As can be observed in **Figure 3**, the extract presented significant anticoagulant activity, prolonging the clotting time in aPTT test almost 3 times, showing that the extract could act on both ophidic and endogenous thrombin. Prolongation of aPTT indicates the inhibition of the intrinsic and/or common pathway [27]. No effect was observed in PT, as shown in **Figure 3**. Considering that the clotting factors activators induce the production of endogenous thrombin as well as the fact that heparin (anticoagulant drug) is able to inhibit the blood incoagulability produced by FX activators toxins from *B. jararaca*, a hypothesis that could be raised is that the extract, indirectly, also inhibits the procoagulant effects produced by clotting factors activators, decreasing the endogenous thrombin produced and thus attenuating the consumption coagulopathy.

In fact, the aqueous leaf extract of *J. gossypiifolia*, in both doses tested (50, 100 and 200 mg/kg), was able to inhibit the defibrinogenating action of *B. jararaca* venom *in vivo* (**Table 2**), corroborating with the results obtained *in vitro*. It was observed that the animals that received only venom presented incoagulable blood even after 60 min of the blood collection. On the other hand, the animals that received venom but were treated with extract by oral route restored the clot capacity, showing results similar to control animals (which received only PBS), thus showing the inhibitory effect of the extract.

**Table 2:** Inhibition of defibrinogenant activity of *B. jararaca* by aqueous leaf extract of *J. gossypiifolia*.

Extract (p.o.)	Venom (i.p.)	Clot formation after 60 min from collection
0 mg/kg (PBS)	0 µg (PBS)	Present
0 mg/kg (PBS)	10 µg	Absence (blood incoagulability)
Jg 50 mg/kg	10 µg	Present
Jg 100 mg/kg	10 µg	Present
Jg 200 mg/kg	10 µg	Present

Results (presence or absence of clot formation) observed in all animals from each group (n = 4/group).

**Figure 4:**
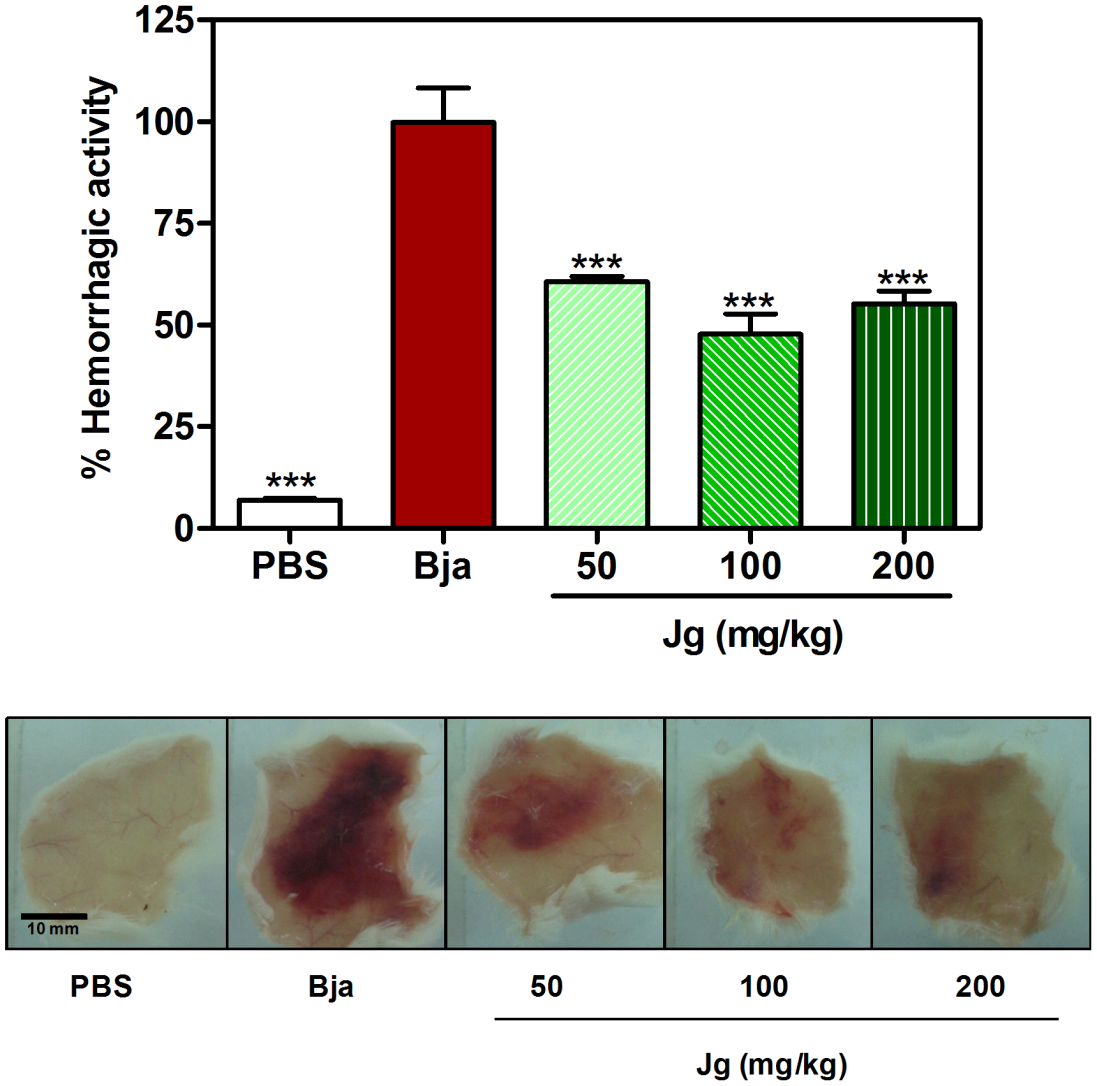
Inhibition of hemorrhagic activity of *B. jararaca* by aqueous leaf extract of *J. gossypiifolia*. *B. jararaca* venom (Bja) was injected s.c. in the dorsal region of animals treated with different p.o. doses of aqueous leaf extract of *J. gossypiifolia* (Jg). 3 h later, the inner surface skin was exposed, photo documented and processed for hemoglobin extraction. A group that received Bja s.c. and PBS p.o. was used as venom control (Bja). Another group that received PBS s.c. and p.o was used as negative control (PBS). In the upper panel, the percentage of activity presented was calculated as: [(Hemoglobin content from treated animals ÷ Hemoglobin content from Bja control animals) ×100]. Values expressed as mean±SEM with n = 5. ***p<0.001 when compared to Bja control (100% of activity) by Tukey's test (ANOVA). In the lower panel, the respective skin aspect from the groups is presented.

In addition to investigation of systemic effects inhibition after envenomation, the local effects were also studied. The inhibition of local hemorrhagic effect of *B. jararaca* venom by aqueous leaf extract of *J. gossypiifolia* was evaluated by quantification of hemoglobin content in the skin injected with venom. As shown in **Figure 4**, the extract, by oral route, clearly attenuated the level of hemorrhage produced by venom. In fact, the hemoglobin content was significantly decreased in treated groups. The local hemorrhage is associated with the action of hemorrhagic SVMPs (hemorrhagins) that causes proteolysis of basal lamina components of microvessels, with loss of vascular wall integrity, leading to blood extravasation to skin [5], [52]. So, the present result could indicate an inhibitory action upon SVMPs action.

PLA_2_s represent a superfamily of lipolytic enzymes which specifically catalyze the hydrolysis of the ester bond at the sn-2 position of glycerophospholipids, resulting in the generation of fatty acids (arachidonate) and lysophospholipids [53], [54]. Apart from their primary catalytic function, snake venom PLA_2_s often display additional pharmacological activities that may be independent of catalytic activity. Hemorrhagic, myotoxic, hemolytic, edematogenic and neurotoxic activities, among others, are described for PLA_2_, implicating the action of PLA_2_s in many of the pharmacological effects seen in snake envenomation [53]. Several studies have demonstrated that the PLA_2_ from *Bothrops* venoms are involved in inflammatory responses such as edema, pain, leukocyte migration, necrosis and myotoxicity [5], [55]. PLA_2_ may be classified as Asp49 or Lys49 PLA_2_, depending on the residue at position 49 in the amino acid sequence. Asp49 PLA_2_s are enzymatically active whereas Lys49 PLA_2_s show little or no enzyme activity, although both types are biologically active [54], [55].

In the phospholipase activity tested in the present study, obviously, only the function of Asp49 PLA_2_s were evaluated. In the experiment, the aqueous leaf extract of *J. gossypiifolia* was inactive (results not shown). In the higher concentration tested (1∶200 w/w, venom: extract), the extract inhibited the phospholipase activity at about 15%, however, this inhibition was not statistically significant (p>0.05). One possible hypothesis is that the extract could present more affinity to enzymatically inactive phospholipases, i.e., Lys49 PLA_2_. So, the *in vivo* edematogenic and myotoxic activities were also investigated.

Bothropic envenomation is characterized by the rapid development of edema and inflammation at the site of venom inoculation [5]. The intraplantar injection of *B. jararaca* venom induces an edematogenic response that appears to be mediated primarily by cyclooxygenase and lypoxygenase eicosanoid products and, in minor extent, evolves the participation of histamine, serotonin and platelet-activating factor (PAF) [56]. Leucocytes exert an important role in the inflammatory response produced in paw edema, being capable of secreting endogenous pro-inflammatory mediators relevant to the acute inflammation development [57]. However, in contrast with other local effects that are produced by direct action of specific toxins, the edematogenic activity is, apparently, a result of a combined action of diverse toxins (Asp49 or Lys49 PLA_2_ and hemorrhagic or non-hemorrhagic SVMPs), rapidly inducing the release of endogenous inflammatory mediators [58], [59]. In fact, their modulation by endogenous mediators often reduces the effectiveness of antivenom, since it is capable of neutralizing the toxins, but cannot reduce inflammation caused by chemical mediators released by these toxins [5].

**Figure 5:**
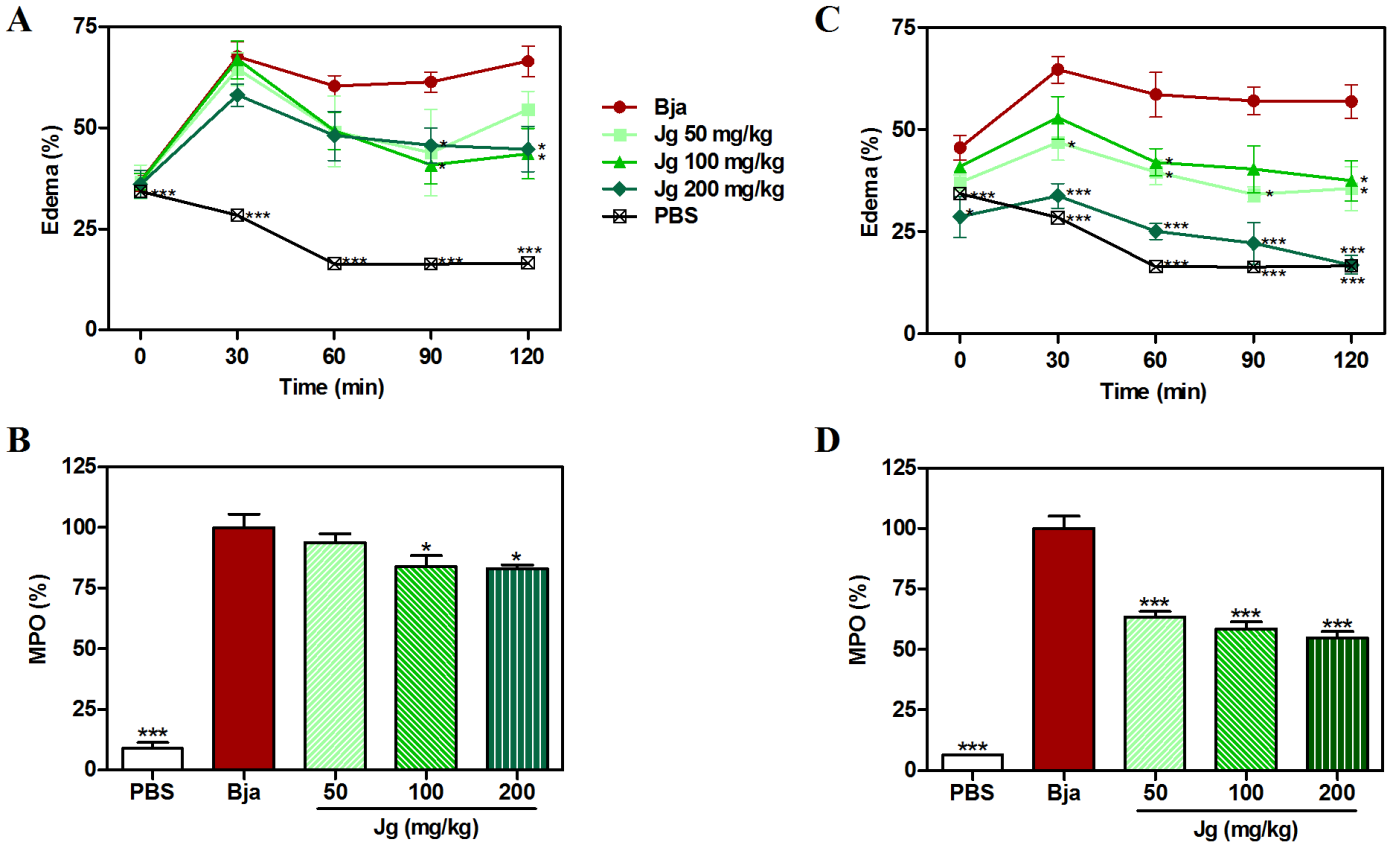
Inhibition of edematogenic activity of *B. jararaca* by aqueous leaf extract of *J. gossypiifolia*. *B. jararaca* venom (Bja) was injected i.pl. in the right hind paw of animals treated with different p.o. or i.p. doses of aqueous leaf extract of *J. gossypiifolia* (Jg). The right hind paw thickness was further measured at 0, 30, 60, 90 and 120 min after injection. A group that received Bja i.pl. and PBS p.o. or i.p. was used as venom control (Bja). Another group that received PBS i.pl. and p.o. or i.p. was used as negative control (PBS). Edema was expressed as the difference between the thickness of the paw after (at respective time) and before (basal values) venom injection, calculated as follows: [(thickness time “t” – thickness before injection) ÷ thickness before injection] ×100 and is presented in **A** (p.o.) and **C** (i.p.). At the end of the experiment, the paws were processed for myeloproxidase (MPO) enzyme extraction. The MPO activity was calculated as: [(MPO from treated animals ÷ MPO from Bja control animals) ×100] and is presented in **B** (p.o.) and **D** (i.p.). Values expressed as mean±SEM with n = 5. *p<0.05 and ***p<0.001 when compared to Bja control (100% of activity) by Tukey's test (ANOVA).

Regarding our results with *J. gossypiifolia* extract, it was observed that the extract, by p.o. route, inhibited the edematogenic response by about 40% after 120 min of the venom injection (**Figure 5A**). As can be observed in **Figure 5A**, the extract was active at 100 and 200 mg/kg, with the inhibitory effect starting from 90 min after venom injection. Regarding MPO activity, in the same way, the extract was active at 100 and 200 mg/kg, showing about 20% inhibition of the MPO activity (**Figure 5B**). With i.p. treatment, even better results were obtained by the extract, as could be observed by the complete inhibition of the edematogenic activity after 120 min of venom injection (**Figure 5C**). By i.p. route, the extract was able to reduce the MPO activity by about 50% (**Figure 5D**). The different activity of the extract in both routes used is expected, since they have different biodisponibility profiles. This significant inhibition of MPO activity could indicate that the antiedematogenic effect presented by the extract could be related to an inhibition of cell migration, since this enzyme is a quantitative marker of inflammatory cell influx to paw tissue injected with venom [32]. Regarding the possible mechanism by which the extract could be acting in this model, some hypothesis could be taken into account based on the experimental results obtained. Once the extract was inactive against Asp49 PLA_2_, a possible inhibition upon Lys49 PLA_2_ could be pointed out. Another possibility may be an inhibitory action upon SVMPs, since the extract also presented anti-hemorrhagic effect, as discussed previously. Additionally, another plausible hypothesis is that the extract could present potent anti-inflammatory activity and, so, may be able to reduce the inflammation produced by endogenous chemical mediators released by toxins. In fact, some studies have shown that anti-inflammatory drugs could inhibit the edematogenic response induced by *B. jararaca* venom [60]. Additionally, some studies have shown the anti-inflammatory activity of *J. gossypiifolia* leaf and aerial parts extracts in various models on inflammation [61]–[63].

**Figure 6:**
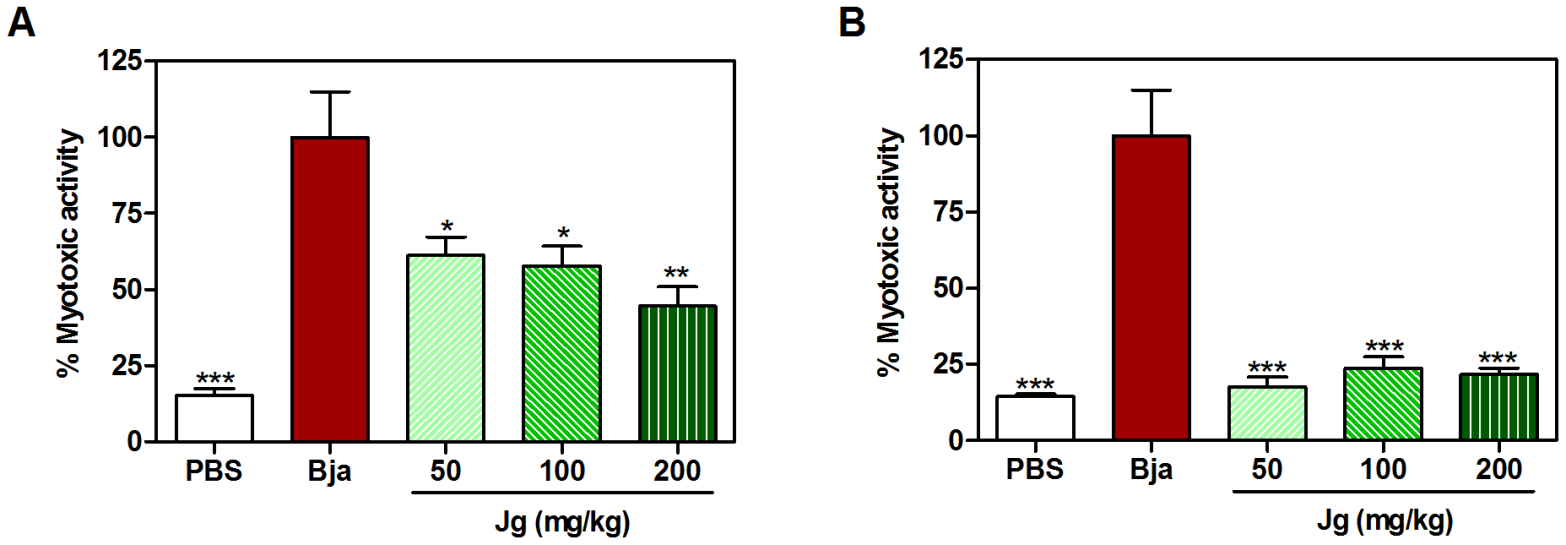
Inhibition of myotoxic activity of *B. jararaca* by aqueous leaf extract of *J. gossypiifolia.* * B. jararaca* venom (Bja) was injected i.m. in the left thigh of animals treated with different p.o. (A) or i.p. (B) doses of aqueous leaf extract of *J. gossypiifolia* (Jg). 3 h later, the blood was collected for creatine kinase (CK) determination. A group that received Bja i.m. and PBS p.o. or i.p. was used as venom control (Bja). Another group that received PBS i.m. and p.o. or i.p. was used as negative control (PBS). The percentage of activity was calculated as: [(CK from treated animals ÷ CK from Bja control animals) × 100]. Values expressed as mean±SEM with n = 5. *p<0.05 and ***p<0.001 when compared to Bja control (100% of activity) by Tukey's test (ANOVA).

Muscle necrosis is a relevant local effect induced by bothropic venom, as it may lead to permanent tissue loss, disability and amputation [55]. The myotoxicity may occur due to a direct rupture action to muscle cells (PLA_2_ toxins) or to an indirect action, which involve the development of ischemia resulting from drastic alteration induced by SVMPs hemorrhagic toxins on both microvasculature and intramuscular arteries [5]. The muscle damage results in cellular membrane rupture, leading to rapid release of cytosolic markers, such as creatine kinase (CK) [5], [55]. In the present work, as can be observed in **Figure 6**, the aqueous leaf extract of *J. gossypiifolia*, by both p.o. and i.p. routes, was able to decrease the CK levels of animals injected with *B. jararaca* venom, which indicates an anti-myotoxic action. Interestingly, by i.p. route, the anti-myotoxic effect reached almost 100%, since the lowest dose. An inhibitory action against Lys49 PLA_2_ could be suggested. Another hypothesis, maybe the most likely according to our results, is that alternatively or complementarily to an action upon Lys49 PLA_2_, the extract while inhibiting the hemorrhagic activity, could reduce the indirect myotoxic activity produced by hemorrhagic SVMPs. In fact, animals from treated groups, when compared with control (treated with PBS), showed in the muscle region, where the venom was injected, a highly significant reduction of hemorrhage (results not shown), which could support the idea that inhibition of hemorrhagic activity was important to reduce muscle damage.

In summary, the obtained results show the potentiality of aqueous leaf extract of *J. gossypiifolia* in the treatment of systemic and also local effects produced by *B. jararaca*, which, in turn, is not efficiently inhibited by conventional antivenom serum therapy. **Table 3** summarizes the inhibition percentage of the extract in the hemorrhagic, edematogenic and myotoxic activities induced by *B. jararaca* injection.

**Table 3:** Maximum percentage of inhibition of *J. gossypiifolia* against local effects produced by *B. jararaca*.

Activity	Dose, route	Inhibition (%)
**Hemorrhagic**	100 mg/kg, p.o.	56.1
Edematogenic*	200 mg/kg, i.p.	99.9
**Myotoxic**	50 mg/kg, i.p.	96.5

Percentage of inhibition was calculated as: [1 – (%activity of test mean –%activity of PBS control mean) ÷ (%activity of Bja control mean –%activity of PBS control mean)] ×100. *In the case of edematogenic activity, the last time point (120 min) of the paw thickness was considered.

In general, the mechanism by which medicinal plants neutralize the toxic effects of snake venoms is still unknown, but many hypothesis have been proposed, such as protein precipitation, proteolytic degradation, enzyme inactivation, metal chelation, antioxidant action or a combination of these mechanisms [11].

According to our results, it could be observed that the extract did not present proteolytic action upon proteins, including the venom, since no change was observed in the electrophoretic pattern of these proteins, except for the gradual vanishing of the bands with the increasing concentrations of extract, which could suggest a protein precipitating action (results not shown). Accordingly, the Bradford dye-protein binding assay was performed to analyze this possible protein precipitating action of the extract. As can be observed in **Table 4**, the aqueous leaf extract of *J. gossypiifolia* was able to precipitate both albumin and *B. jararaca* venom proteins, in both tested concentrations. The precipitating percentage reached was almost 100% upon venom proteins. Thus, the obtained results could explain, at least partially, the inhibitory effects presented by aqueous leaf extract of *J. gossypiifolia*.

**Table 4:** Precipitating activity of aqueous leaf extract of *J. gossypiifolia*.

Albumin/Bja: Jg (w/w)	% Protein precipitation	
	Upon albumin	Upon *B. jararaca* venom
**1∶1**	67.5±0.9	78.2±1.4
**1∶50**	74.5±1.6	97.1±0.3
**1∶100**	82.4±0.8	99.0±0.6

Bja: *B. jararaca* venom. Jg: aqueous leaf extract of *J. gossypiifolia.*

Values expressed as mean±SEM with n = 3.

Another activity investigated in aqueous leaf extract of *J. gossypiifolia* was its antioxidant potential. The snake venom causes intense inflammation and local tissue damage, inducing macrophages and neutrophils to generate reactive oxygen species such as superoxide radicals, which act by forming lipid peroxides which could cause necrosis [64]. It is considered, in general, that lipid peroxidation inhibition could be beneficial against toxic effects produced by snake venom and that the antioxidant process could be an important mechanism by which antiophidic molecules could act [11], [64]. In the present work, the aqueous leaf extract of *J. gossypiifolia* presented significant antioxidant activity in all tested *in vitro* models (**Table 5**). The extract was able to chelate metal ions (copper and iron), scavenge free radicals (hydroxyl and superoxide) and presented reducing power. Therefore, a possible hypothesis is that the antiophidic activity presented by the extract could be related, at least in part, to its ability to inhibit the oxidative stress induced by envenomation and consequently indirectly inhibit the necrosis process. Similar observations have been made by other authors when evaluating the antiophidic and antioxidant activities of an extract from the plant *Crinum jagus* [65].

**Table 5:** Antioxidant effects of aqueous leaf extract of *J. gossypiifolia*.

Activity	Jg	Standard
**Total antioxidant capacity** *	155.2±13.36 AAE/g	-
**Copper chelation**	IC_50_ = 0.111 µg/µL	IC_50_ = 0.017 µg/µL (EDTA)
**Iron chelation**	IC_50_ = 1.104 µg/µL	IC_50_ = 0.011 µg/µL (EDTA)
**Hydroxyl radical scavenging**	IC_50_ = 1.205 µg/µL	IC_50_ = 0.293 µg/µL (gallic acid)
**Superoxide radical scavenging**	IC_50_ = 0.118 µg/µL	IC_50_ = 0.003 µg/µL (gallic acid)
**Reducing power** * **^#^**	Concentration producing equivalent activity to 0.2 µg/µL ascorbic acid = 0.581 µg/µL	-

Jg: aqueous leaf extract of *J. gossypiifolia.* AAE/g: mg ascorbic acid equivalent per gram of extract.

*Value expressed as mean±SEM with n = 3.

The chelating action presented by the extract in antioxidant assays is very interesting, since several toxins need metals as essential cofactors for their enzymatic activity, as for example SVMPs, which are metalloproteinases zinc-dependent. Metal chelating agents, such as EDTA or *o*-phenanthroline, inhibit the action of these toxins [66]. So, a hypothesis that could be pointed out is that one possible mechanism of the SVMPs inhibition presented by *J. gossypiifolia* aqueous leaf extract is metal chelation. In fact, a recent study showed that glycolic acid, a potent antioxidant and metal chelator agent, was able to inhibit the enzymatic and biological effects of the SVMP BaP1, a metalloproteinase isolated from *Bothrops asper* snake venom [67].

In summary, the results presented in this paper demonstrate the potentiality of aqueous leaf extract of *J. gossypiifolia* as an antiophidic agent, with inhibitory action upon hemostatic and inflammatory effects induced by *B. jararaca* venom. It is important to note that the administration route used (oral route) simulates the popular use of the plant as a tea, showing that this product could be, at least, a good palliative to snakebite until the specific treatment can be started, considering that the antivenom, in many cases, can be hard to access. Another point of view is that the tea of the leaves of *J. gossypiifolia* could be utilized as an adjuvant to antivenom serum therapy. In either case, the use of the plant could be advantageous in view of its ability to reverse systemic and local effects and its easy availability and low cost inherent to its preparation. An overview of the results obtained in the present paper is summarized in **Figure S2**.

Further studies aiming the isolation of the biological active compounds of the crude extract, especially flavonoids, are underway in our research group. Additionally, as a future perspective to better understand the inhibitory effect shown by aqueous leaf extract of *J. gossypiifolia*, we intend to study thoroughly the interaction between bioactive compounds isolated from the extract with toxins isolated from *B. jararaca* venom, e.g. SVMPs and Lys49 PLA_2_.

In conclusion, the results demonstrate the potential antiophidic activity of *J. gossypiifolia* aqueous leaf extract, including its significant action upon local effects, suggesting that this species may be used as a new source of bioactive molecules against bothropic venom, especially for the treatment of venom local effects.

## Supporting Information

Figure S1 ***Jatropha gossypiifolia*** **L. (Euphorbiaceae) plant.** Photography by Juliana Félix-Silva.
(DOCX)

Figure S2 **Overview of antiophidic activity of aqueous leaf extract of** ***J. gossypiifolia*** **against** ***B. jararaca*** **venom.** *Bothrops jararaca* snake venom induces systemic and local effects in victim of envenoming. As could be observed by the inhibition of azocaseinolytic, fibrinogenolytic and defibrinogenating activity inhibition, as well as by the anticoagulant activity presented in activated partial thromboplastin time (aPTT) test, the aqueous leaf extract of *Jatropha gossypiifolia* was able to inhibit the systemic effect of blood incoagulability produced by *B. jararaca* venom. Besides inhibiting this systemic effect, the extract was able to efficiently inhibit the local effects produced, as could be observed by the inhibition of edematogenic, hemorrhagic and myotoxic activities *in vivo*. It is important to note that the *in vivo* inhibitory actions was achieved by intraperitoneal and oral administration of the extract, which is interesting to be pointed since the oral route simulates the popular use of the plant as a tea. Regarding possible toxicity, the extract was evaluated by *in vitro* methods of cytotoxicity, using human embryonic kidney cells (HEK-293) and red blood cells (RBC) and absence of toxicity was observed, suggesting a possible low toxicity of the extract. The phytochemical analysis revealed the presence of alkaloids, terpenes and/or steroids, phenolic compounds, flavonoids, tannins and amines.
(DOCX)

## References

[1] Gutiérrez JM, Theakston DG, Warrell DA (2006) Confronting the neglected problem of snake bite envenoming: the need for a global partnership. PLoS Med 3: 727–731. 10.1371/journal.pmed.0030150 PMC1472552 16729843

[2] Kasturiratne A, Wickremasinghe AR, Silva N, Gunawardena NK, Pathmeswaran A, et al (2008) The global burden of snakebite: a literature analysis and modelling based on regional estimates of envenoming and deaths. PLoS Med 5: 1591–1604. 10.1371/journal.pmed.0050218 PMC2577696 18986210

[3] Gutiérrez JM, Warrell DA, Williams DJ, Jensen S, Brown N, et al (2013) The need for full integration of snakebite envenoming within a global strategy to combat the neglected tropical diseases: the way forward. PLoS Negl Trop Dis 7: e2162. 23785526 10.1371/journal.pntd.0002162 PMC3681653

[4] Ministério da Saúde (2009) Guia de Vigilância Epidemiológica. Caderno 14. Brasília: Secretaria de Vigilância em Saúde.

[5] Gutiérrez JM, Lomonte B (1989) Local tissue damage induced by *Bothrops* snake venoms. A review. Mem Inst Butantan 51: 211–223.

[6] Kang TS, Georgieva D, Genov N, Murakami MT, Sinha M, et al (2011) Enzymatic toxins from snake venom: structural characterization and mechanism of catalysis. FEBS J 278: 4544–4576. 21470368 10.1111/j.1742-4658.2011.08115.x

[7] Gutiérrez JM, León G, Burnouf T (2011) Antivenoms for the treatment of snakebite envenomings: the road ahead. Biologicals 39: 129–142. 21429763 10.1016/j.biologicals.2011.02.005

[8] Mors WB, Nascimento MC, Ruppelt Pereira BM, Alvares Pereira N (2000) Plant natural products active against snake bite - the molecular approach. Phytochemistry 55: 627–642. 11130675 10.1016/s0031-9422(00)00229-6

[9] Santhosh MS, Hemshekhar M, Sunitha K, Thushara RM, Jnaneshwari S, et al (2013) Snake venom induced local toxicities: plant secondary metabolites as an auxiliary therapy. Mini Rev Med Chem 13: 106–123. 22876950

[10] Dey A, De JN (2012) Phytopharmacology of antiophidian botanicals: a review. Int J Pharmacol 8: 62–79.

[11] Gomes A, Das R, Sarkhel S, Mishra R, Mukherjee S, et al (2010) Herbs and herbal constituents active against snake bite. Indian J Exp Biol 48: 865–878. 21506494

[12] Albuquerque UP, Monteiro JM, Ramos MA, de Amorim ELC (2007) Medicinal and magic plants from a public market in northeastern Brazil. J Ethnopharmacol 110: 76–91. 17056216 10.1016/j.jep.2006.09.010

[13] Di Stasi LC, Hiruma-Lima CA (2002) Plantas medicinais na Amazônia e na Mata Atlântica. São Paulo: UNESP. 604 p.

[14] Félix-Silva J, Giordani RB, Silva Jr AA, Zucolotto SM, Fernandes-Pedrosa MF (2014) *Jatropha gossypiifolia* L. (Euphorbiaceae): a review of traditional uses, phytochemistry, pharmacology, and toxicology of this medicinal plant. Evid Based Complement Alternat Med 2014: 1–32. 10.1155/2014/369204 PMC4070477 25002902

[15] Brasil (2011) RENISUS: Relação Nacional de Plantas Medicinais de Interesse ao SUS. Espécies vegetais. Ministério da Saúde. Available: http://portal.saude.gov.br/portal/arquivos/pdf/RENISUS.pdf. Accessed 2011 Aug 7.

[16] Wagner H, Bladt S (2001) Plant drug analysis: a thin layer chromatography atlas. Germany: Springer. 384 p.

[17] Dubois M, Gilles KA, Hamilton JK, Rebers PA, Smith F (1956) Colorimetric method for determination of sugars and related substances. Anal Chem 28: 350–356.

[18] Melo-Silveira RF, Fidelis GP, Costa MSSP, Telles CBS, Dantas-Santos N, et al (2012) *In vitro* antioxidant, anticoagulant and antimicrobial activity and in inhibition of cancer cell proliferation by xylan extracted from corn cobs. Int J Mol Med Sci 13: 409–426. 10.3390/ijms13010409 PMC3269695 22312261

[19] Bradford MM (1976) A rapid and sensitive method for the quantitation of microgram quantities of protein utilizing the principle of protein-dye binding. Anal Biochem 72: 248–254. 942051 10.1016/0003-2697(76)90527-3

[20] Robert S, Baccelli C, Devel P, Dogné J-M, Quetin-Leclercq J (2010) Effects of leaf extracts from *Croton zambesicus* Müell. Arg. on hemostasis. J Ethnopharmacol 128: 641–648. 20219668 10.1016/j.jep.2010.02.007

[21] Mossmann T (1983) Rapid colorimetric assay for cellular growth and survival: application to proliferation and cytotoxicity assays. J Immunol Methods 65: 55–63. 6606682 10.1016/0022-1759(83)90303-4

[22] Moura LDA, Sanchez EF, Bianco ÉM, Pereira RC, Teixeira VL, et al (2011) Antiophidian properties of a dolastane diterpene isolated from the marine brown alga *Canistrocarpus cervicornis* . Biomed Prev Nutr 1: 61–66. 10.1016/j.biopha.2010.09.023 21131161

[23] Rodrigues VM, Soares AM, Guerra-Sá R, Rodrigues V, Fontes MRM, et al (2000) Structural and functional characterization of neuwiedase, a nonhemorrhagic fibrin(ogen)olytic metalloprotease from *Bothrops neuwiedi* snake venom. Arch Biochem Biophys 381: 213–224. 11032408 10.1006/abbi.2000.1958

[24] Laemmli UK (1970) Cleavage of structural proteins during the assembly of the head of bacteriophage T4. Nature 227: 680–685. 5432063 10.1038/227680a0

[25] Feitosa L, Gremski W, Veiga SS, Elias MCQB, Graner E, et al (1998) Detection and characterization of metalloproteinases with gelatinolytic, fibronectinolytic and fibrinogenolytic activities in Brown spider (*Loxosceles intermedia*) venom. Toxicon 36: 1039–1051. 9690796 10.1016/s0041-0101(97)00083-4

[26] Theakston RDG, Reid HA (1983) Development of simple standard assay procedures for the characterization of snake venoms. Bull World Health Org 61: 949–956. 6609011 PMC2536230

[27] Mao W, Li H, Li Y, Zhang H, Qi X, et al (2009) Chemical characteristic and anticoagulant activity of the sulfated polysaccharide isolated from *Monostroma latissimum* (Chlorophyta). Int J Biol Macromol 44: 70–74. 19007806 10.1016/j.ijbiomac.2008.10.003

[28] Melo KRT, Camara RBC, Queiroz MF, Vidal AAJ, Lima CRM, et al (2013) Evaluation of sulfated polysaccharides from the brown seaweed *Dictyopteris justii* as antioxidant agents and as inhibitors of the formation of calcium oxalate crystals. Molecules 18: 14543–14563. 24287990 10.3390/molecules181214543 PMC6269805

[29] Habermann E, Hardt KL (1972) A sensitive and specific plate test for the quantitation of phospholipases. Anal Biochem 50: 163–173. 4342994 10.1016/0003-2697(72)90495-2

[30] Roodt AR, Dolab JA, Dokmetjian JC, Litwin S, Segre L, et al (2000) A comparison of different methods to assess the hemorrhagic activity of *Bothrops* venoms. Toxicon 38: 865–873. 10695971 10.1016/s0041-0101(99)00205-6

[31] Maiorano VA, Marcussi S, Daher MAF, Oliveira CZ, Couto LB, et al (2005) Antiophidian properties of the aqueous extract of *Mikania glomerata* . J Ethnopharmacol 102: 364–370. 16084045 10.1016/j.jep.2005.06.039

[32] Bradley PP, Priebat DA, Christensen RD, Rothstein G (1982) Measurement of cutaneous inflammation: estimation of neutrophil content with an enzyme marker. J Invest Dermatol 78: 206–209. 6276474 10.1111/1523-1747.ep12506462

[33] Mebs D, Ehrenfeld M, Samejima Y (1983) Local necrotizing effect of snake venoms on skin and muscle: relationship to serum creatine kinase. Toxicon 21: 393–404. 6623487 10.1016/0041-0101(83)90096-x

[34] Núñez V, Otero R, Barona J, Saldarriaga M, Osorio RG, et al (2004) Neutralization of the edema-forming, defibrinating and coagulant effects of *Bothrops asper* venom by extracts of plants used by healers in Colombia. Braz J Med Biol Res 37: 969–977. 15264003 10.1590/s0100-879x2004000700005

[35] Ambikabothy J, Ibrahim H, Ambu S, Chakravarthi S, Awang K, et al (2011) Efficacy evaluations of *Mimosa pudica* tannin isolate (MPT) for its anti-ophidian properties. J Ethnopharmacol 137: 257–262. 21640180 10.1016/j.jep.2011.05.013

[36] Zhang XP, Zhang ML, Su XH, Huo CH, Gu YC, et al (2009) Chemical constituents of the plants from genus *Jatropha* . Chem Biodivers 6: 2166–2183. 20020449 10.1002/cbdv.200700461

[37] Pilon AC, Carneiro RL, Carnevale Neto F, Bolzani VS, Castro-Gamboa I (2013) Interval multivariate curve resolution in the dereplication of HPLC-DAD data from *Jatropha gossypifolia* . Phytochem Anal 24: 401–406. 23483597 10.1002/pca.2423

[38] Subramanian SS, Nagarajan S, Sulochana N (1971) Flavonoids of the leaves of *Jatropha gossypiifolia* . Phytochemistry 10: 1690–1690.

[39] Franke K, Nasher AK, Schmidt J (2004) Constituents of *Jatropha unicostata* . Biochem Sys Ecol 32: 219–220.

[40] Kuppusamy UR, Das NP (1993) Protective effects of tannic acid and related natural compounds on *Crotalus adamenteus* subcutaneous poisoning in mice. Pharmacol Toxicol 72: 290–295. 8372049 10.1111/j.1600-0773.1993.tb01652.x

[41] Devappa RK, Makkar HPS, Becker K (2010) *Jatropha* toxicity - a review. J Toxicol Environ Health B Crit Rev 13: 476–507. 20711929 10.1080/10937404.2010.499736

[42] Mariz SR, Cerqueira GS, Araújo WC, Dantas JG, Ramalho JA, et al (2012) Chronic toxicologic study of the ethanolic extract of the aerial parts of *Jatropha gossypiifolia* in rats. Rev Bras Farmacogn 22: 663–668.

[43] Nagaharika Y, Kalyani V, Rasheed S (2013) Ramadosskarthikeyan (2013) Anti-inflammatory activity of leaves of *Jatropha gossypifolia* L. by HRBC membrane stabilization method. JAD 2: 156–158.

[44] Sabandar CW, Ahmat N, Jaafar FM, Sahidin I (2013) Medicinal property, phytochemistry and pharmacology of several *Jatropha* species (Euphorbiaceae): a review. Phytochemistry 85: 7–29. 23153517 10.1016/j.phytochem.2012.10.009

[45] De Paula RC, Sanchez EF, Costa TR, Martins CHG, Pereira PS, et al (2010) Antiophidian properties of plant extracts against *Lachesis muta* venom. J Venom Anim Toxins Incl Trop Dis 16: 311–323.

[46] Vilar JC, Carvalho CM, Furtado MFD (2007) Effects of the aqueous extracts of plants of the genera *Apodanthera* (Cucurbitaceae) and *Jatropha* (Euphorbiaceae) on the lethality of the venom of *Bothrops jararaca* (serpentes, Viperidae). Biol Geral Exper 7: 32–39.

[47] Sajevic T, Leonardi A, Križaj I (2011) Haemostatically active proteins in snake venoms. Toxicon 57: 627–645. 21277886 10.1016/j.toxicon.2011.01.006

[48] Bjarnason JB, Fox JW (1994) Hemorrhagic metalloproteinases from snake venoms. Pharmacol Therap 62: 325–372. 7972338 10.1016/0163-7258(94)90049-3

[49] Kornalík F (1985) The influence of snake venom enzymes on blood coagulation. Pharmacol Ther 29: 353–405. 3915360 10.1016/0163-7258(85)90008-7

[50] Swenson S, Markland Jr FS (2005) Snake venom fibrin(ogen)olytic enzymes. Toxicon 45: 1021–1039. 15882884 10.1016/j.toxicon.2005.02.027

[51] Stocker K, Fischer H, Meier J (1982) Thrombin-like snake venom proteinases. Toxicon 20: 265–273. 7043783 10.1016/0041-0101(82)90225-2

[52] Escalante T, Rucavado A, Fox JW, Gutiérrez JM (2011) Key events in microvascular damage induced by snake venom hemorrhagic metalloproteinases. J Proteomics 74: 1781–1794. 21447411 10.1016/j.jprot.2011.03.026

[53] Arni RK, Ward RJ (1996) Phospholipase A_2_ - a structural review. Toxicon 34: 827–841. 8875770 10.1016/0041-0101(96)00036-0

[54] De Paula RC, Castro HC, Rodrigues CR, Melo PA, Fuly AL (2009) Structural and pharmacological features of phospholipases A_2_ from snake venoms. Protein Pept Lett 16: 899–907. 19689416 10.2174/092986609788923365

[55] Gutiérrez JM, Lomonte B (1995) Phospholipase A_2_ myotoxins from *Bothrops* snake venoms. Toxicon 33: 1405–1424. 8744981 10.1016/0041-0101(95)00085-z

[56] Trebien HA, Calixto JB (1989) Pharmacological evaluation of rat paw oedema induced by *Bothrops jararaca* venom. Agents Actions 26: 292–300. 2660497 10.1007/BF01967293

[57] Cury Y, Teixeira CFP, Sudo LS (1994) Edematogenic responses induced by *Bothrops jararaca* venom in rats: role of lymphocytes. Toxicon 32: 1425–1431. 7886700 10.1016/0041-0101(94)90414-6

[58] Gutiérrez JM, Rucavado A (2000) Snake venom metalloproteinases: their role in the pathogenesis of local tissue damage. Biochimie 82: 841–850. 11086214 10.1016/s0300-9084(00)01163-9

[59] Teixeira CFP, Landucci ECT, Antunes E, Chacur M, Cury Y (2003) Inflammatory effects of snake venom myotoxic phospholipases A_2_ . Toxicon 42: 947–962. 15019493 10.1016/j.toxicon.2003.11.006

[60] Araújo SD, De Souza A, Nunes FPB, Gonçalves LRC (2007) Effect of dexamethasone associated with serum therapy on treatment of *Bothrops jararaca* venom-induced paw edema in mice. Inflamm Res 56: 409–413. 18026697 10.1007/s00011-007-7054-x

[61] Félix-Silva J, Gomes JAS, Barbosa LMQ, Pinheiro ITMG, Soares LAL, et al (2014) Systemic and local anti-inflammatory activity of aqueous leaf extract from *Jatropha gossypiifolia* L. (Euphorbiaceae). Int J Pharm Pharm Sci 6: 142–145.

[62] Bhagat R, Ambavade SD, Misar AV, Kulkarni DK (2011) Anti-inflammatory activity of *Jatropha gossypifolia* L. leaves in albino mice and wistar rat. J Sci Ind Res 70: 289–292.

[63] Panda BB, Gaur K, Kori ML, Tyagi LK, Nema RK, et al (2009) Anti-inflammatory and analgesic activity of *Jatropha gossypifolia* in experimental animal models. Global Journal of Pharmacology 3: 1–5.

[64] Shenoy PA, Nipate SS, Sonpetkar JM, Salvi NC, Waghmare AB, et al (2013) Anti-snake venom activities of ethanolic extract of fruits of *Piper longum* L. (Piperaceae) against Russell's viper venom: characterization of piperine as active principle. J Ethnopharmacology 147: 373–382. 10.1016/j.jep.2013.03.022 23506990

[65] Ode OJ, Nwaehujor CO, Onakpa MM (2010) Evaluation of antihaemorrhagic and antioxidant potentials of *Crinum jagus* bulb. IJABPT 1: 1330–1336.

[66] Ramos OHP, Selistre-De-Araujo HS (2006) Snake venom metalloproteases - structure and function of catalytic and disintegrin domains. Comp Biochem Physiol C Pharmacol Toxicol Endocrinol 142: 328–346. 10.1016/j.cbpc.2005.11.005 16434235

[67] Pereañez JA, Patiño AC, Rey-Suarez P, Núñez V, Henao Castañeda IC, et al (2013) Glycolic acid inhibits enzymatic, hemorrhagic and edema-inducing activities of BaP1, a P–I metalloproteinase from *Bothrops asper* snake venom: insights from docking and molecular modeling. Toxicon 71: 41–48. 23726855 10.1016/j.toxicon.2013.05.013

