# Supplementary material for: Aqueous Leaf Extract of Jatropha gossypiifolia L. (Euphorbiaceae) Inhibits Enzymatic and Biological Actions of Bothrops jararaca Snake Venom
Source: PLoS One. 2014 Aug 15;9(8):e104952. doi: 10.1371/journal.pone.0104952 (PMC4134247; doi:10.1371/journal.pone.0104952)
Supplement: Figure S1 — Jatropha gossypiifolia L. (Euphorbiaceae) plant. Photography by Juliana Félix-Silva. (DOCX) [file pone.0104952.s001.docx]

**Supplementary Figure 1.**

**
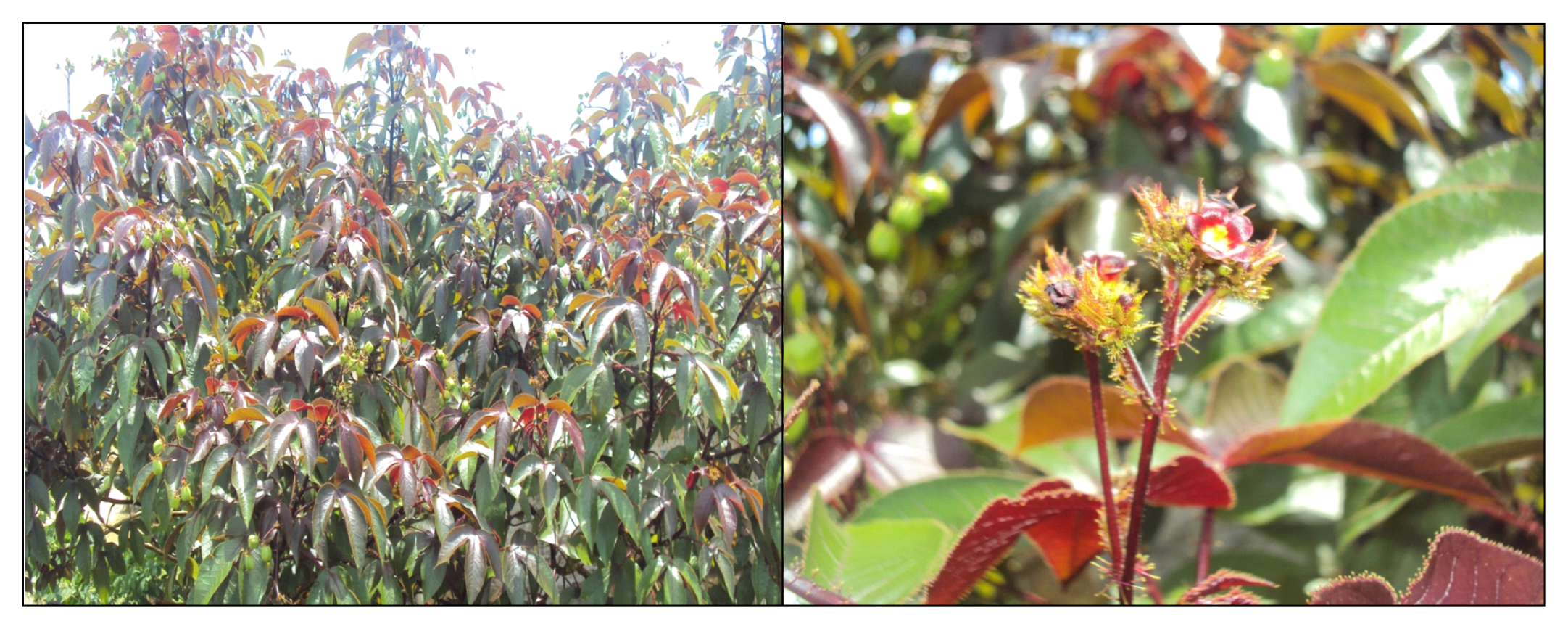
**

***Jatropha gossypiifolia* L. (Euphorbiaceae) plant at the site of collection.** Photography by Juliana Félix-Silva.
