# Supplementary material for: Aqueous Leaf Extract of Jatropha gossypiifolia L. (Euphorbiaceae) Inhibits Enzymatic and Biological Actions of Bothrops jararaca Snake Venom
Source: PLoS One. 2014 Aug 15;9(8):e104952. doi: 10.1371/journal.pone.0104952 (PMC4134247; doi:10.1371/journal.pone.0104952)
Supplement: Figure S2 — Overview of antiophidic activity of aqueous leaf extract of J. gossypiifolia against B. jararaca venom. Bothrops jararaca snake venom induces systemic and local effects in victim of envenoming. As could be observed by the inhibition of azocaseinolytic, fibrinogenolytic and defibrinogenating activity inhibition, as well as by the anticoagulant activity presented in activated partial thromboplastin time (aPTT) test, the aqueous leaf extract of Jatropha gossypiifolia was able to inhibit the systemic effect of blood incoagulability produced by B. jararaca venom. Besides inhibiting this systemic effect, the extract was able to efficiently inhibit the local effects produced, as could be observed by the inhibition of edematogenic, hemorrhagic and myotoxic activities in vivo. It is important to note that the in vivo inhibitory actions was achieved by intraperitoneal and oral administration of the extract, which is interesting to be pointed since the oral route simulates the popular use of the plant as a tea. Regarding possible toxicity, the extract was evaluated by in vitro methods of cytotoxicity, using human embryonic kidney cells (HEK-293) and red blood cells (RBC) and absence of toxicity was observed, suggesting a possible low toxicity of the extract. The phytochemical analysis revealed the presence of alkaloids, terpenes and/or steroids, phenolic compounds, flavonoids, tannins and amines. (DOCX) [file pone.0104952.s002.docx]

**Supplementary Figure 2.**
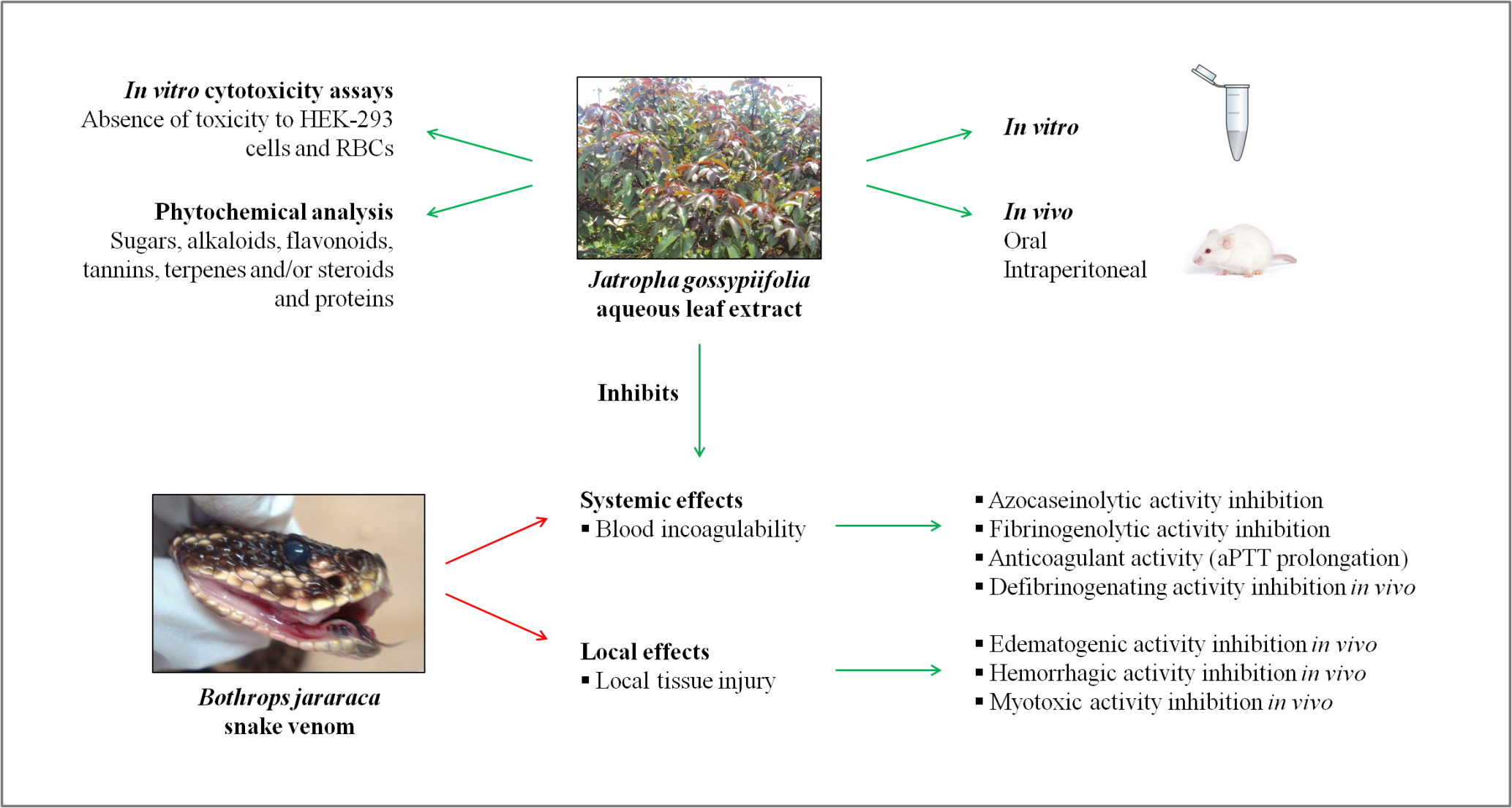


**Overview of antiophidic activity of aqueous leaf extract of *J. gossypiifolia* against *B. jararaca* venom.** *Bothrops jararaca* snake venom induces systemic and local effects in victim of envenoming. As could be observed by the inhibition of azocaseinolytic, fibrinogenolytic and defibrinogenating activity inhibition, as well as by the anticoagulant activity presented in activated partial thromboplastin time (aPTT) test, the aqueous leaf extract of *Jatropha gossypiifolia* was able to inhibit the systemic effect of blood incoagulability produced by *B. jararaca* venom. Besides inhibiting this systemic effect, the extract was able to efficiently inhibit the local effects produced, as could be observed by the inhibition of edematogenic, hemorrhagic and myotoxic activities *in vivo*. It is important to note that the *in vivo* inhibitory actions was achieved by intraperitoneal and oral administration of the extract, which is interesting to be pointed since the oral route simulates the popular use of the plant as a tea. Regarding possible toxicity, the extract was evaluated by *in vitro* methods of cytotoxicity, using human embryonic kidney cells (HEK-293) and red blood cells (RBC) and absence of toxicity was observed, suggesting a possible low toxicity of the extract. The phytochemical analysis revealed the presence of alkaloids, terpenes and/or steroids, phenolic compounds, flavonoids, tannins and amines.
